# Functional insights into nucleoside diphosphate kinases encoded by two *ndk* paralogs in *Waddlia chondrophila*

**DOI:** 10.1016/j.crmicr.2026.100635

**Published:** 2026-06-17

**Authors:** Giti Ghazi-Soltani, Carole Kebbi-Beghdadi, Simone E. Adams, Gilbert Greub

**Affiliations:** Center for Research on Intracellular Bacteria, Institute of Microbiology, University Hospital Center and University of Lausanne, Lausanne, Switzerland

**Keywords:** *Waddlia chondrophila*, Nucleoside diphosphate kinase (Ndk), AZT inhibition, Subcellular localization, Protein trafficking

## Abstract

The *Chlamydiota* phylum consists of obligate intracellular bacteria, including well-known pathogens and emerging environmental species, with diverse host ranges and metabolic capabilities. Among these bacteria, the gene which encodes nucleoside diphosphate kinase, *ndk* is present in variable numbers. While most chlamydial species carry a single paralog of *ndk*, some species have two paralogs. In Waddlia chondrophila, the two Ndk proteins encoded by *ndk* paralogs retain conserved kinase motifs but differ in subcellular localization, suggesting divergent functional roles. According to localization studies performed in heterologous expression systems, WcNdk1 is confined to the bacteria-containing vacuole and probably supports nucleotide metabolism, while WcNdk2 localizes to the host nucleus, perinuclear space, and Golgi apparatus, suggesting involvement in host interaction. Azidothymidine (AZT), a known Ndk inhibitor, impaired W. chondrophila growth, potentially through inhibition of WcNdk2. However, the lack of genetic tools currently limits definitive functional conclusions. Our data suggests potential functions for Ndks in *W. chondrophila,* providing a foundation for future studies on Ndk-mediated interactions between this pathogen and its host, which might possibly be translated into a new therapeutic approach.

## Highlights

• W. chondrophila encodes two Ndk paralogs, unlike most Chlamydiota.
• Ndk genes are early expressed and protein levels peak during bacterial replication.
• WcNdk1 is inclusion-bound, while WcNdk2 localizes to host cell compartments.
• AZT inhibits bacterial growth and protects host cells from infection-induced death.
• AZT sensitivity is linked to ndk2 presence, implicating WcNdk2 as functional target.

## Graphical abstract

**Figure:**
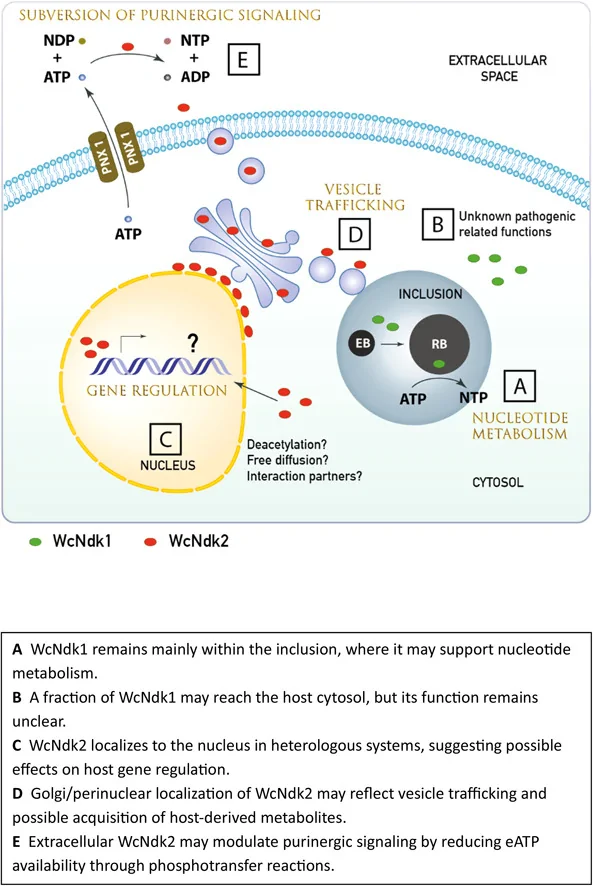

## 1 Introduction

The *Chlamydiota* phylum encompasses several families of obligate intracellular bacteria, including six main families: *Chlamydiaceae, Parachlamydiaceae, Waddliaceae, Simkaniaceae, Rhabdochlamydiaceae*, and *Criblamydiaceae*. Members of these families share a biphasic developmental cycle, which reflects their adaptation to intracellular environments and reliance on host cell machinery for survival.

*Waddlia chondrophila* belongs to the family *Waddliaceae* and is known as an abortigenic agent in ruminants (Kebbi-Beghdadi et al., 2026). It was first isolated from an aborted bovine fetus (Dilbeck et al., 1990; Henning et al., 2002) and serological studies showed a strong association between anti-*W. chondrophila* antibodies and bovine abortion (Dilbeck-Robertson et al., 2003). In humans, *W. chondrophila* seropositivity is significantly associated with adverse pregnancy outcome (Baud et al., 2007, 2008, 2011). This pathogen was also detected in respiratory tract samples of patients with pneumonia and children with bronchiolitis (Goy et al., 2009; Haider et al., 2008).

The *W. chondrophila* developmental cycle is biphasic and is divided into three major phases (de Barsy and Greub, 2013; Kebbi-Beghdadi et al., 2011). In the early phase (0–8 h post-infection, hpi) elementary bodies (EBs) enter host cells and reside in a vacuole, termed an inclusion, where they differentiate into reticulate bodies (RBs). This phase is followed by a proliferation phase (8-24 hpi) during which the number of RBs increases exponentially through binary fission. In the late phase (24-48 hpi), RBs revert into EBs, and lysis of the host cell releases infectious progeny. Under iron deprivation, environmental (heat shock) or antibiotic stress, the developmental cycle is arrested, leading to an enlarged, non-dividing RB-like form called aberrant body (AB) (Kozusnik et al., 2025; Rodriguez Rozo et al., 2026; Scherler et al., 2020). While *W. chondrophila* significantly impacts both animal and human health, the specific molecular and genomic pathways, particularly those involving developmental and regulatory proteins, remain largely unexplored. Since nucleoside diphosphate kinases (Ndks) of *W. chondrophila* are secreted in the cytosol of infected eukaryotic cells (Kebbi-Beghdadi et al., 2019; 2020), we were interested in characterizing their function, to better understand the biology of this bacterium and its adaptation inside host cells.

Ndk is a highly conserved enzyme present in both bacterial and eukaryotic cells, where it contributes to nucleotide pool homeostasis by catalyzing reversible γ-phosphate transfer between nucleoside triphosphates and nucleoside diphosphates through a conserved phospho-histidine intermediate (Adam et al., 2020; Boissan et al., 2018; Kapoor & Varshney, 2020). Balanced NTP and dNTP pools are essential for DNA replication, genome stability, growth and cellular fitness (Kapoor & Varshney, 2020; Nordman & Wright, 2008; Schaaper & Mathews, 2013).

Beyond its housekeeping functions, Ndk is implicated in various cellular processes, including cell differentiation and development in eukaryotes and prokaryotes (Masoudi et al., 2013; Muñoz-Dorado et al., 1990; Timmons & Shearn, 2000; Wang et al., 2019). Additionally, Ndk is involved in the regulation of gene expression (Kumar et al., 2005; Levit et al., 2002; Postel et al., 2000), as well as in the modulation of cellular physiology in cyanobacteria (Kirti & Rajaram, 2025). Although Ndk is classically described as an intracellular enzyme, extracellular forms have been reported in several bacterial pathogens (Bunce & Khanim, 2018; Kolli et al., 2008; Yilmaz et al., 2008). In this extracellular context, Ndk can decrease extracellular ATP (eATP) availability by using ATP as a phosphate donor in phosphotransfer reactions, thereby potentially modulating eATP-dependent purinergic signaling and inflammatory responses (Bunce & Khanim, 2018; Coutinho-Silva & Ojcius, 2012; Kolli et al., 2008; Yilmaz et al., 2008).

We previously reported transcriptomic data for *W. chondrophila* comparing genes expression in RBs at 24 hpi and EBs at 72 hpi (Ardissone et al., 2020). In that dataset, both *ndk* genes were differentially expressed between the two developmental stages, each showing a fold change of 0.3 in EBs compared with RBs. Despite the well-established functions of Ndk across various organisms, its specific function in the *Chlamydiota* members remains uncharacterized. Based on these observations, we hypothesized that this enzyme could play an important role in the development and pathogenesis of *W. chondrophila*. In this study, we characterize the two Ndk proteins in this organism to better understand their potential role in the bacterium’s development. This work provides an overall view of Ndk proteins in the representatives of *Chlamydiota* phylum and offers functional insights into the Ndks proteins of *W. chondrophila*. Our results pave the way for more detailed research once genetic tools become available for *W. chondrophila*.

## 2 Material and methods

### 2.1 Phylogenetic and structural analysis

Species-based and *ndk* gene phylogenies were retrieved from the *Chlamydia* Database (Pillonel et al., 2020). Trees were trimmed using FigTree (v1.4.4) to retain only relevant *Chlamydiota* species. The Ndk protein sequences across different taxa were retrieved from the National Center for Biotechnology Information (NCBI). Multiple sequence alignment of Ndk proteins across eukaryotes and prokaryotes was performed using the ClustalW tool in UGENE v1.30.0. The corresponding similarity heat map (percentage identity, excluding gap) was also generated on UGENE. Alignment visualization was carried out with ESPript https://espript.ibcp.fr/ESPript/cgi-bin/ESPript.cgi. The 3D structure of the protein was reconstructed using Phyre2 (Kelley et al., 2015) and visualized using Jmol v.16.2.15.

### 2.2 Cell culture and *W. chondrophila* infection

McCoy cells (murine fibroblast, ATCC CRL-1696, purchased from ATCC, USA), HEK293T cells (human embryonic kidney, ATCC CRL-11268, kind gift from Dr. T. Roger, Lausanne University Hospital) or HeLa cells (human cervical adenocarcinoma epithelial cells, ATCC CCL-2, kind gift from Dr. T. Roger) were maintained at 37°C in 5% CO_2_ in DMEM GlutaMAX (Thermo Fisher Scientific, USA), supplemented with 10% fetal bovine serum (FBS) (Thermo Fisher Scientific, USA). *W. chondrophila* (ATCC VR-1471) was propagated in *Acanthamoeba castellanii* (ATCC 30010) at 25°C in T-25 flasks containing 6 ml of peptone–yeast–extract–glucose broth. At the time of infection, lysed *W. chondrophila*-infected amoebae were filtered through a 5 µm syringe filter to remove amoebal debris. The bacterial solution was then used to infect the host cells at a dilution which was optimum for infection (MOI 0.1–1). To synchronize the infection, infected cells were centrifuged at 1790 g for 10 min. They were then incubated at 37°C in 5% CO_2_ for 15 min. Following incubation, the inoculum was then replaced with fresh medium.

### 2.3 Purification of recombinant his-tagged WcNdk1 and WcNdk2 for antiserum production

The *E. coli* strain BL21 containing pET28a-*wcndk1-6xHis* or pET28a-*wcndk2-6xHis* was grown to an OD_600_ of 0.5. The culture was induced using 1 mM isopropyl-β-D-thiogalactopyranoside (IPTG) (Applichem, Germany) and incubated at 37°C for 4 h to express the recombinant protein. Bacterial pellets were resuspended in native lysis buffer (50 mM NaH_2_PO_4_, 300 mM NaCl, and 10 mM imidazole, pH 8). The resuspended cells were lysed using a combination of methods: three cycles of freeze (ethanol-dry ice bath) and thaw (at 37°C), followed by chemical lysis with 1 mg/ml lysozyme (AppliChem, Germany) and sonication. The recombinant protein was purified under native condition using Ni-NTA agarose beads (Qiagen, Germany) according to the manufacturer’s instructions. Briefly, bacterial lysates were incubated with Ni-NTA resin for 2 h at 4°C with gentle rotation. The protein-bound resin was then loaded onto poly-prep chromatography columns (Bio-Rad, USA), washed with wash buffer (50 mM Tris-HCl, 300 mM NaCl, 20 mM imidazole, pH 8.0), and eluted with the same buffer containing 250 mM imidazole. The purified protein was dialyzed using Slide-A-Lyzer Dialysis Cassette, 2000 MWCO (Thermo Scientific, USA) overnight against PBS to remove imidazole. The concentration of the protein was determined using Bradford’s reagent with bovine serum albumin (Bio-Rad, USA) as a standard. Following protein purification, rabbit and mouse polyclonal antisera were produced using immunization services offered by Eurogentec SA (Seraing, Belgium).

### 2.4 Western blot

McCoy cells were seeded at a density of 1 × 10^6^ per T-25 flask one day before infection and infected with *W. chondrophila* as described above. Infected cells were harvested by scraping at the specified hpi and a fraction of the culture was saved for extraction and quantification of the genomic DNA (see below). The cell suspension was pelleted at 1790 g for 10 min, then washed twice with PBS, and finally resuspended in 0.5 ml of 1 x Laemmli sample buffer (Bio-Rad, USA). An equal volume of each lysate was resolved on 12% SDS-PAGE precast gels (Bio-Rad, USA) and transferred onto an Amersham Protran nitrocellulose membrane (Cytiva, USA). The membrane was blocked in saturation buffer (10 mM Tris-Base, 150 mM NaCl, and 0.05% Tween 20) containing 5% non-fat dried milk (AppliChem, Germany) for 2 h at room temperature. Blots were probed overnight at 4°C with antibodies against WcNdk1 and WcNdk2 diluted 1/500 and 1/200 in saturation buffer with 0.5% non-fat dried milk. After three washes with saturation buffer containing 0.5% non-fat dried milk, blots were incubated with a 1/3000 dilution of secondary antibodies (horseradish peroxidase-conjugated anti-rabbit IgG (Promega, USA) or anti-mouse IgG (Bio-Rad, USA)) for 1 h at room temperature and processed using the Amersham ECL detection system (Cytiva, USA). Western blot bands intensities were quantified using EvolutionCapt edge software (Vilber, France) and normalized to the corresponding bacterial genome copy number. Graphs were generated using GraphPad v. 10.4.1. Uncropped images of all blots are shown in the Supplementary Fig. S3.

### 2.5 Extraction of genomic DNA and quantification of *W. chondrophila* genome copy number

Genomic DNA from collected samples was extracted using the Wizard SV Genomic DNA purification system (Promega, USA) according to the manufacturer’s instructions. The extracted DNA served as a template for qPCR with iTaq Universal Probes Supermix (Bio-Rad, USA) to quantify the *W. chondrophila* bacterial population. The *16S rRNA* gene copy numbers were determined using a standard curve, which was generated from serial dilutions of a plasmid containing one copy of the *16S rRNA* gene. The primer sequences (Goy et al., 2009) are listed in Supplementary Table S1.

### 2.6 RNA extraction and cDNA synthesis

*W. chondrophila-*infected McCoy cell monolayers or uninfected control cells were harvested at indicated hpi by scraping and centrifugation (5000g, 10 min). Following centrifugation, cells were lysed directly in TRIzol Reagent (Invitrogen, USA). Total RNA was extracted by chloroform separation and recovery of the aqueous phase (12,000g, 15min, 4°C). RNA was precipitated with isopropanol, washed with 75% ethanol, air-dried, and resuspended in RNase-free water. To remove genomic DNA, samples were treated with RNase-free DNaseI (Invitrogen, USA) following the manufacturer’s instructions. cDNA was synthesized using the GoScript Reverse Transcription System (Promega, USA) with random primers. Reverse transcription was performed at 42°C for 60 min, followed by enzyme inactivation at 70°C for 15 min. All cDNAs were diluted 1:5 prior to qPCR.

### 2.7 RT-qPCR

The qPCR reactions were set up in a final volume of 20 μl containing 4 μl of cDNA template, the appropriate concentration of each primer, and 1x iTaq Universal SYBR Green Supermix (Bio-Rad, USA). All samples were run in duplicates and no-template controls were included for each primer pair to assess non-specific amplification. The fluorescent reporter signal was normalized against the internal reference dye (ROX) signal. qPCR was carried out on QuantStudio 3 Real-Time PCR System (Applied Biosystems, USA) using the following thermal program. A single cycle of DNA polymerase activation for 3 min at 95°C, followed by 45 amplification cycles of 15 sec at 95°C (denaturing step) and 1 min at 60 C (annealing and extension step). Relative gene expression was calculated using the comparative 2^^-ΔΔCt^ method (Livak & Schmittgen, 2001). Transcript levels of target genes were normalized to the *16S rRNA* gene, used as an internal reference standard for bacterial gene expression (Bustin et al., 2009) and the 24hpi time point was used as reference sample. Quantitative data from qRT-PCR experiments were collected from at least three independent replicates. The primers used for RT-qPCR are listed in Supplementary Table S1.

### 2.8 Application of AZT on *W. chondrophila* infected cells and cell death assessment

McCoy cells were seeded at a density of 1 × 10^4^ cells per well in 96-well plates (Corning, USA). Cells were infected with *W. chondrophila* or left uninfected as a control. To monitor cell death, 7 µg/ml propidium iodide (PI) (Sigma-Aldrich, Germany) was added to the growth medium. AZT (3′-azido-3′-deoxythymidine) was purchased from Tocris, (Bio-Techne, Switzerland) and a 10 mM stock solution was prepared by dissolving 50 mg in 18.7 ml water (pH was not checked). AZT was added to the wells at concentrations ranging from 0 to 250 µg/ml in six technical replicates per condition. Depending on the experimental design, AZT was added at the time of infection (0 hpi) or at later time points (3, 8, 24, 32, and 48 hpi). PI fluorescence was measured at specified time points post-infection using a FLUOstar Omega plate reader (BMG LABTECH, Germany) (excitation: 540 nm; emission: 640 nm). To define 100% cell death, 0.1% Triton X-100 was added to control wells prior to PI reading.

### 2.9 Antibodies, immunofluorescence assay, and confocal microscopy

Mouse anti-V5 antibody was purchased from Thermo Fisher Scientific (USA). Goat anti-Chlamydia trachomatis major outer membrane protein (MOMP) antibodies were obtained from Lifespan Bioscience (USA). Polyclonal antibodies against W. chondrophila, E. lausannensis, and S. negevensis were homemade. Mouse antibodies against GM130 (a cis-Golgi marker) and rabbit anti-tubulin antibodies were obtained from BD Biosciences (USA) and Abcam (UK), respectively. Secondary anti-mouse anti-rabbit or anti-goat antibodies conjugated to Alexa Fluor 488 or 594, as well as Texas Red–conjugated concanavalin A, were purchased from AppliChem (Germany). DAPI (4′,6-diamidino-2-phenylindole) was purchased from Thermo Fisher Scientific (USA).

McCoy cells were cultured on glass coverslips placed in 24-well plates and infected with various bacterial species. At the indicated time points, cells were fixed with ice-cold methanol or 4% paraformaldehyde (PFA) for 5 min at 4°C, followed by three washes with phosphate-buffered saline (PBS). Fixed cells were permeabilized and blocked for 30 min using a blocking solution containing 0.1% saponin, 10% FBS, and 0.04% sodium azide in PBS. Blocked cells were incubated with primary antibodies for 2 h at room temperature (anti-V5 diluted 1:5000, anti-MOMP diluted 1:500 and anti GM130 diluted 1:300). After three washing steps with blocking solution, cells were incubated for 1 h with Alexa Fluor 488- or 594-conjugated secondary antibodies (1:1000 dilution) to label bacteria. DAPI (1:3000 dilution) and concanavalin A (1:50 dilution) were used to stain nuclei and carbohydrates, respectively. Cells were then washed three times with PBS and briefly rinsed with purified water before mounting. Coverslips were mounted on glass slides with Mowiol 4-88 (Sigma-Aldrich, USA) and stored in the dark until imaging using Zeiss LSM 900 confocal laser scanning microscope (Zeiss, Germany).

For Supplementary Fig. S1, rabbit anti-WcNdk1 antibody was used at a dilution of 1:500 on methanol-fixed cells. Mouse anti-WcNdk2 antibody was used at a dilution of 1:500 on PFA-fixed cells, followed by permeabilization with 1% Triton X-100 in PBS for 20 min at room temperature. For antibody specificity assessment in transfection experiments (Supplementary Fig. S5), both antibodies were used at a 1:500 dilution. Anti-WcNdk1 showed no detectable cross-reactivity under either fixation condition (PFA or methanol). Anti-WcNdk2 showed some cross-reactivity under methanol fixation; however, no cross-reactivity was observed under PFA fixation conditions, which were used for the experiments shown in Supplementary Fig. S1.

### 2.10 Subcellular fractionation

McCoy cells infected with *C. trachomatis* expressing WcNdk2-V5 were harvested by scraping and centrifuged at 500g for 10 min. The cell pellet was washed twice with PBS (500g, 10 min). Cells were then resuspended in 500 μl of freshly prepared Buffer A (10 mM HEPES pH 7.9, 10 mM KCl, 0.1 mM EDTA, 0.1 mM EGTA) supplemented with protease inhibitors (Thermo Fisher Scientific, USA) and incubated for 10 min at room temperature. NP-40 (Merck, Switzerland) was added to a final concentration of 0.2%, and cells were mechanically disrupted by five passages through a 27G needle. Samples were centrifuged at 800g for 5 min and the supernatant was collected as the cytosolic fraction after a second spin (800g, 5 min). The nuclear pellet was washed twice with Buffer A (800g, 5 min).

For extraction of the nuclear soluble fraction, the pellet was resuspended in RIPA buffer (Sigma-Aldrich, USA) supplemented with 2 mM EDTA and protease inhibitors (Thermo Fisher Scientific, USA), incubated for 30 min at room temperature, and centrifuged at 16,000 g for 10 min. The supernatant was collected as the nuclear soluble fraction. The remaining pellet, corresponding to the nuclear non-soluble (chromatin-associated) fraction, was resuspended in 1 × Laemmli sample buffer.

### 2.11 pGL2 and pBOMBL plasmid construction

The genes *wcndk1* and *wcndk2* were amplified from *W. chondrophila* genomic DNA using primers with 5′ overhangs compatible with the pGL2 (a kind gift from the Scott Hefty laboratory, University of Kansas, USA) or pBOMBL (generously provided by Scot Ouellette laboratory, University of Nebraska Medical Center, USA) backbone. All PCR products included a C-terminal V5 epitope tag to facilitate detection. PCR products were purified using the QIAquick PCR Purification Kit (Qiagen, Germany). The pBOMBL vector was linearized with EagI and KpnI (NEB, USA). The native *C. trachomatis*–derived pGL2 vector (11786 bp; β-lactamase selection marker) was linearized by AgeI digestion (NEB, USA). Both vectors were recovered from agarose gels with the QIAquick Gel Extraction Kit (Qiagen, Germany). Purified inserts and linearized vectors were assembled via In-fusion cloning using 5 x In-fusion Snap assembly Master Mix (Takara Bio, Japan). Assemblies were incubated at 50°C for 15 min. Transformations were performed using *E. coli* dam⁻ dcm⁻ cells. Positive clones were screened by colony PCR, and successful insertions were confirmed by Sanger sequencing using both vector- and insert-specific primers.

### 2.12 *C. trachomatis* transformation

Transformation of *C. trachomatis* was performed with minor modifications to previously described methods (Mueller et al., 2017). Briefly, 1 × 10^6^ McCoy cells were seeded in 6-well plates and cultured overnight. 2.5 × 10^6^ plasmid-free C. trachomatis serovar L2 EBs, were resuspended in 300 µl Tris-CaCl_2_ buffer (10 mM Tris, 50 mM CaCl_2_, pH 7.4) and incubated with 2 µg of sequence-verified plasmid DNA for 30 min at room temperature. 1 ml Hank’s balanced salt solution (HBSS) (Gibco, Thermo Fisher Scientific, USA) was then added to each reaction. This mixture was added to McCoy cells in a 6-well plate after removing the medium. The infection was carried out by centrifugation at 400g for 15 min at room temperature and incubation at 37°C for 15 min. Then, the inoculum was removed, and cells were incubated with 2 ml of DMEM medium supplemented with 10% FBS for 8 h at 37°C and 5% CO₂. After this incubation, the medium was replaced with DMEM containing 10% FBS, 1 µg/ml cycloheximide (Sigma-Aldrich, Germany) and 0.6 mg/ml penicillin G (Sigma-Aldrich, Germany) or 50 µg/ml spectinomycin (Sigma-Aldrich, Germany) to select for transformed bacteria. Cells were passaged every 48 h, and the development of fluorescent inclusions was monitored until they were clearly observed and established. The transformed *C. trachomatis* were harvested and titrated by IFU assay and stored at -80°C. For experiments requiring induction, 50 ng/ml anhydrotetracycline (aTc) (Sigma-Aldrich, Germany) was added at the time of infection, whereas, in control conditions, the inducer was excluded.

### 2.13 Cloning in pDEST47 and pcDNA5 for mammalian expression

For Gateway cloning in pDEST47 expression plasmids, attB-flanked primers were used to amplify *wcndk1, wcndk2* or *wcndk2* without the first 51 nucleotides and genes were tagged with a V5 epitope at their C-terminus. Entry clones were generated by BP recombination using the Gateway BP Clonase II Enzyme Mix (Thermo Fisher Scientific, USA). The resulting pDONR201-*wcndk1-V5*, pDONR201-*wcndk2-V5* and pDONR201-*wcndk2ΔSP-V5* entry clones were selected on kanamycin (AppliChem, Germany) plates. Destination vectors were generated via LR recombination of the entry clones with pDEST47 using the Gateway LR Clonase II Enzyme Mix (Thermo Fisher Scientific, USA) following the supplier’s instructions. To generate the SP-mCherry construct, the nucleotide sequence encoding the predicted WcNdk2 signal peptide (19 aa; MLKKLIFTAAILFAPLFAQ) was fused to the N-terminus of mCherry by PCR amplification using Phusion High-Fidelity DNA Polymerase (NEB, USA). The resulting amplicon and the pcDNA5 vector were digested with KpnI and XhoI (NEB, USA). Digested PCR products and plasmids were purified, and restriction enzymes were heat-inactivated at 80°C for 20 min. The digested insert was ligated into the digested pcDNA5 vector using T4 DNA ligase (NEB, USA). Final constructs were transformed in *E. coli,* purified using the GeneJET Plasmid Miniprep Kit (Thermo Fisher Scientific, USA), and verified by Sanger sequencing before transfection experiments.

### 2.14 Transient transfection of HEK293T and HeLa cells

Glass coverslips placed in 24-well plates were coated with 100 µg/ml poly-L-lysine (Sigma-Aldrich, Germany) for 30 min at room temperature, washed three times with PBS, and air-dried for 3 h. HEK293T cells were seeded on coated coverslips at 2 × 10⁵ cells per well and incubated overnight at 37°C in 5% CO₂. HeLa cells were seeded on uncoated coverslips at 2.5 × 10⁵ cells per well and incubated under the same conditions. Twenty-four hours post-seeding, cells were transfected with pDEST47 expression plasmids using the Lipofectamine 3000 Transfection Kit (Thermo Fisher Scientific, USA). According to the manufacturer’s instructions. Briefly, for each well, 0.5 µg plasmid DNA was diluted in 25 µL serum-free DMEM containing 1 µL P3000 reagent. Separately, 0.75 µL Lipofectamine 3000 reagent was diluted in 25 µL serum-free DMEM. The two mixtures were combined, incubated for 15 min at room temperature, and 50 µL of the transfection complex was added dropwise in each well. Cells were fixed 24 h post-transfection with 4% PFA for downstream immunofluorescence analysis as described above.

## 3 Results

### 3.1 Evolution of the ndk gene cluster in representatives of the *Chlamydiota* phylum

**Fig. 1:**
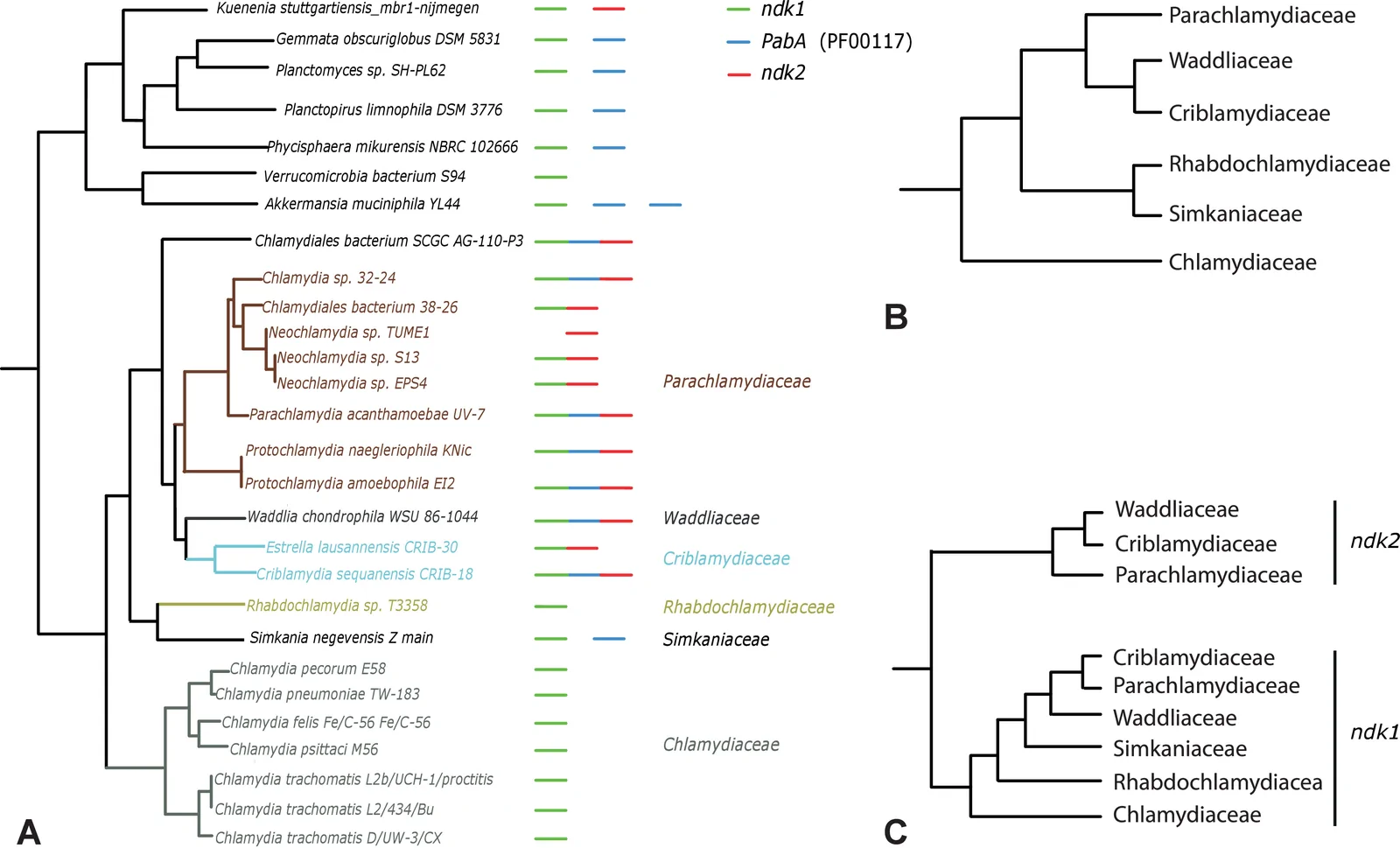
Phylogeny of Chlamydiota phylum and the ndk gene operon evolution. (A) Species phylogeny based on 32 single-copy orthologous genes. (B) Simplified representation of the species phylogeny highlighting major clades. (C) Phylogeny based on the ndk gene, showing its evolutionary relationship among Chlamydial species.

Similar to other organisms, all bacteria of the *Chlamydiota* phylum possess at least one *ndk* gene. A second paralog (*ndk2*) is present in members of the *Parachlamydiaceae, Waddliaceae*, and *Criblamydiaceae* families (Fig. 1A). Interestingly, *ndk2* forms a probable operon with the ancestral *ndk1* gene. In most species, this gene cluster is further associated with *pabA*, which encodes para-aminobenzoate synthase an enzyme involved in the folate biosynthesis pathway. Comparison of gene-based and species-based phylogenetic trees (Fig. 1B and C) suggests that the duplication of *ndk2* most likely originated from a single gene duplication event in the common ancestor of the *Parachlamydiaceae, Waddliaceae*, and *Criblamydiaceae*. However, a scenario of ancestral duplication with subsequent loss in *Simkaniaceae* and *Chlamydiaceae* cannot be excluded, although it is less parsimonious.

### 3.2 High sequence and structural conservation of ndk across species

**Fig. 2:**
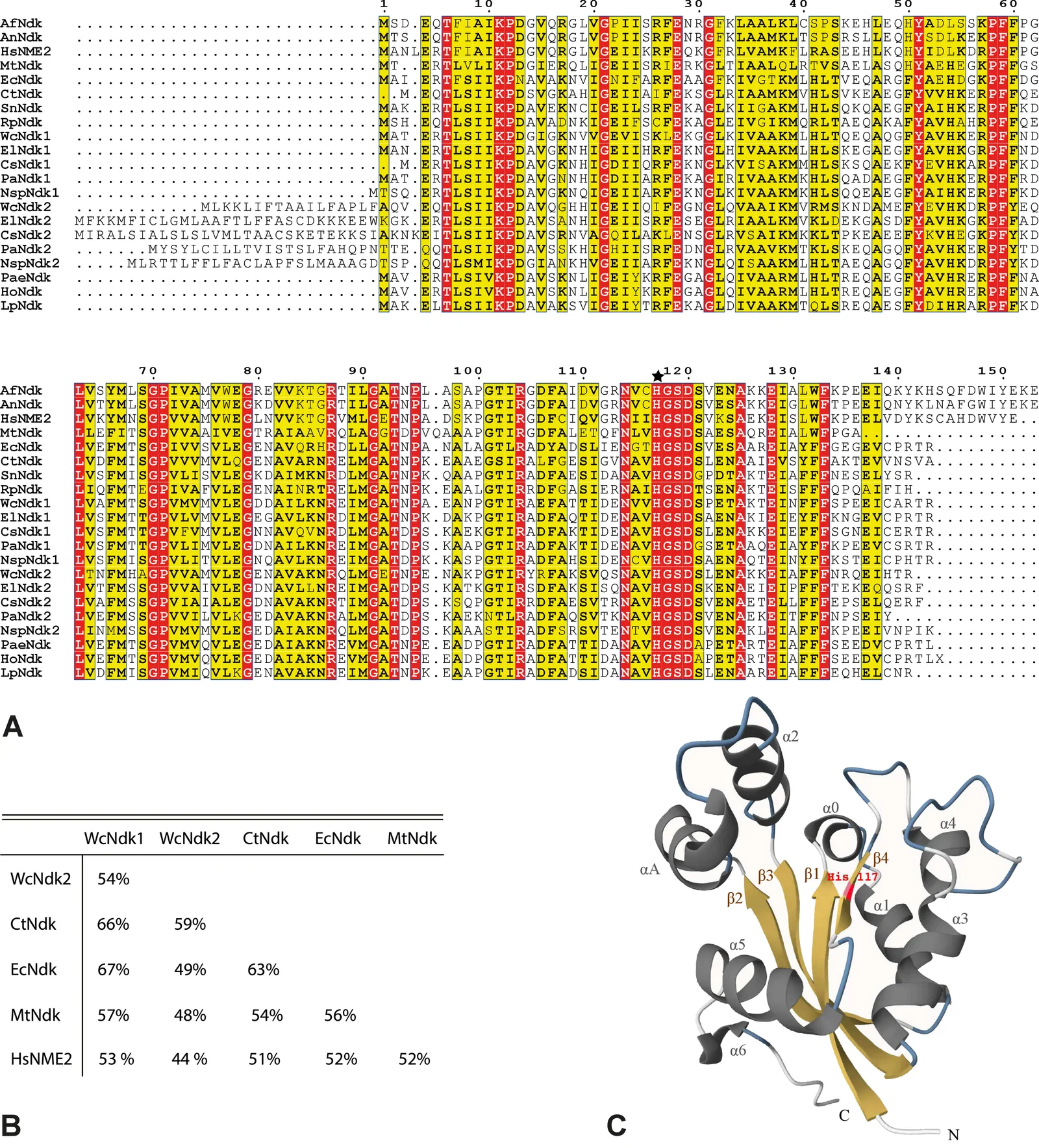
Ndk sequence and structure conservation. (A) Multiple sequence alignment of Ndk proteins across eukaryotes and prokaryotes. Conserved residues are highlighted in red with the highly conserved H residue, essential for catalytic activity, marked with an asterisk. Af: Aspergillus flavus, An: A. niger, Hs: Homo sapiens, Mt: Mycobacterium tuberculosis, Ec: Escherichia coli, Ct: C. trachomatis, Sn: Simkania negevensis, Rp: Rhabdochlamydia porcellionis, Wc: W. chondrophila, El: Estrella lausannensis, Cs: Criblamydia sequanensis, Pa: Parachlamydia acanthamoebae, Nsp: Neochlamydia sp., Pae: Pseudomonas aeruginosa, Ho: Halofilum ochraceum, Lp: Legionella pneumophila (B) Amino acid sequence identity matrix comparing Ndk proteins from W. chondrophila (WcNdk1 and WcNdk2), Chlamydia trachomatis (CtNdk), Escherichia coli (EcNdk), Mycobacterium tuberculosis (MtNdk), and human Ndk (HsNME2). (C) Predicted 3D structure of WcNdk1, illustrating its conserved α/β fold characteristic of the Ndk family. 3D structure reconstructed using Phyre2 and visualized using Jmol v.16.2.15. The H177, highlighted in red, is a critical histidine residue and positioned within the active site.

The multiple sequence alignment shows a high degree of sequence conservation of Ndk proteins from various bacterial and eukaryotic species (Fig. 2A). Specifically, the His-Gly-Ser-Asp (HGSD) motif, which is the enzyme’s active site (Lascu and Gonin, 2000; Arumugam and Ajitkumar, 2012), is well conserved across all aligned sequences. The highly conserved histidine residue inside this motif transiently receives the phosphate group during autophosphorylation reaction and is therefore critical for the catalytic function of Ndk. The Kpn-loop-associated region is also partially conserved across the aligned Ndk homologs, including the core PGTIR motif located upstream of the catalytic HGSD motif (Fig. 2A). In addition to these motifs, other conserved residues are observed in regions associated with nucleotide binding and structural stability, many of which are enriched in hydrophobic amino acids. These residues are likely to contribute to the proper folding and enzymatic efficiency of Ndk.

W. chondrophila genome contains two paralogs of ndk: *wcndk1 (*locus wcw_1543) and *wcndk2* (locus wcw_1545), whose encoded proteins share 54% amino acid sequence identity (Fig. 2B). The most striking difference between these two paralogs is the presence of a predicted signal peptide of 18 amino acids (predicted by SignalP version 6.0 with a probability of 0.999) in WcNdk2, which is absent in WcNdk1.

The sequence identity matrix of *W. chondrophila* Ndk proteins, WcNdk1 and WcNdk2, and their counterparts from other bacterial and eukaryotic species reveals differing degrees of conservation as shown in Fig. 2B. WcNdk1 shares the highest sequence identity with *E. coli* Ndk (EcNdk) and *Chlamydia trachomatis* Ndk (CtNdk) (67% and 66%, respectively). In contrast, WcNdk2 exhibits slightly lower sequence identity across species, which may indicate functional divergence of the two Ndks following gene duplication. Interestingly, both *W. chondrophila* Ndk homologs display relatively high sequence identity (∼50%) with human NME2/NDPK-B/NM23-H2 (HsNME2), which suggests the conservation of core functional domains across vast evolutionary distance. Sequence identity matrix of Ndk proteins across different species is shown in Supplementary Table S2.

WcNdk1 has 143 residues forming a polypeptide chain and adopting a very similar fold to Ndk proteins from other origins, including human Ndk (Singh et al., 2023; Janin et al., 2000; Georgescauld et al., 2020) (Fig. 2C). One Ndk unit has α/β domains comprising a four-stranded antiparallel β-sheet and two connecting α-helices. This high degree of sequence and structural conservation between eukaryotes and pathogens may enable them to interfere with host cellular processes by mimicking host Ndk.

### 3.3 Expression profile of *W. chondrophila* Ndks during infection

Temporal expression levels of genes and biosynthesis of proteins across different developmental stages may help us understand their stage-specific roles and regulatory mechanisms. Since each of these stages has unique biological features, we can indirectly infer a protein’s function by mapping both transcript and protein levels across these developmental stages.

**Fig. 3:**
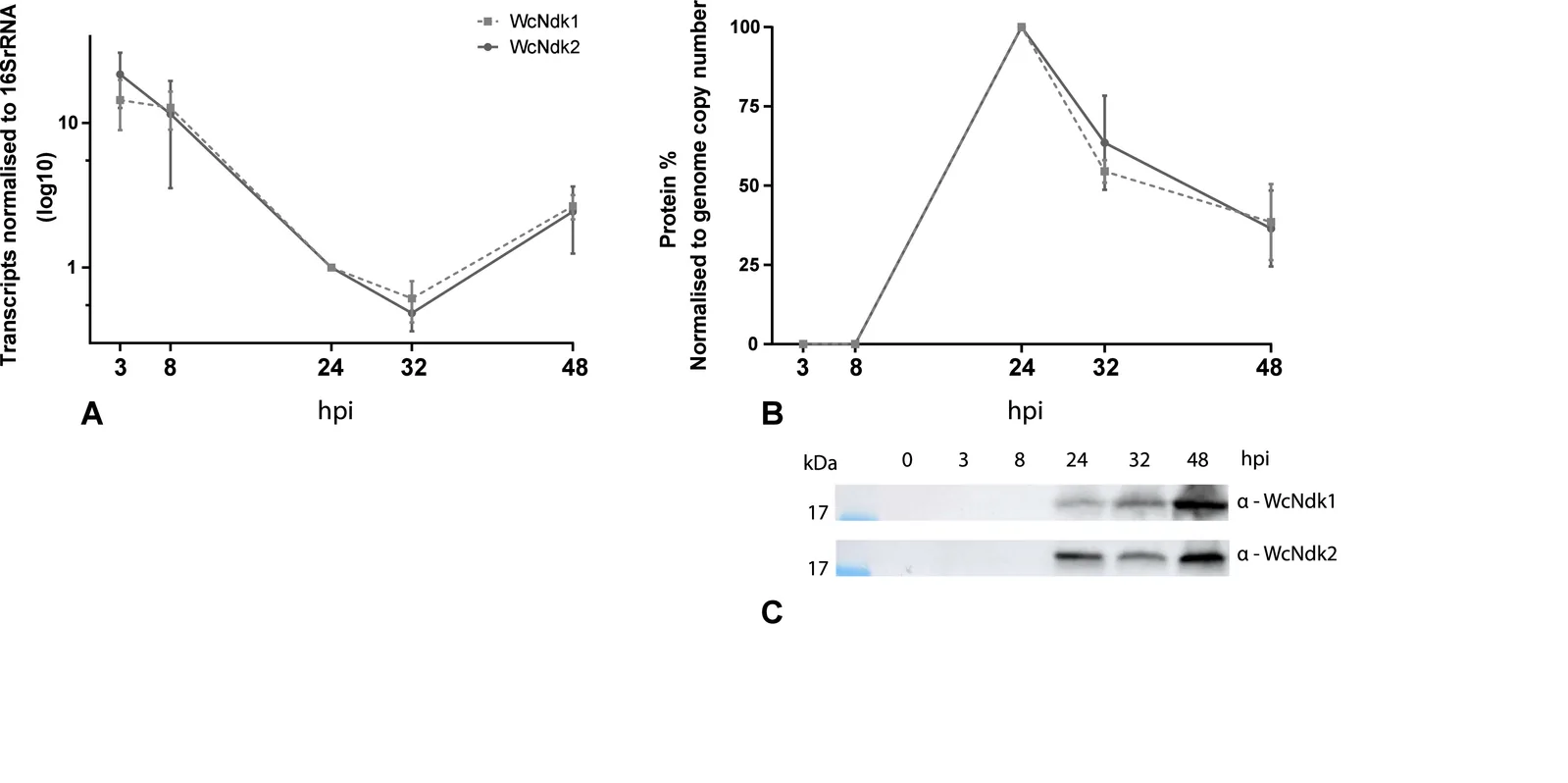
Temporal expression analysis of ndk paralogs in W. chondrophila during infection. (A) Quantitative RT-PCR analysis of ndk1 and ndk2 transcript levels at different hours post-infection (hpi), normalized to 16S rRNA. Transcript abundance is expressed relative to the 24 hpi time point, which was set as the reference (1.0). (B) Relative protein levels of WcNdk1 and WcNdk2 quantified by Western blot, normalized to bacterial genome copy number. Protein expression is shown relative to 24 hpi, set as 100%. (C) Representative Western blot images showing WcNdk1 and WcNdk2 expression across the infection time course. Data are shown as mean ± SD of 3 independent experiments.

We quantified the expression profiles of the *W. chondrophila ndk*s, at both the transcript and protein levels during McCoy cell infection (Fig. 3). Noteworthy, *wcndk1* and *wcndk2* transcripts were highly abundant at early time points (3-8 hpi) as revealed by RT-qPCR, then the mRNA level declined sharply to a minimum at 32 hpi, with a moderate surge toward the end of the cycle (48 hpi) (Fig. 3A). In parallel, protein expression profiles of WcNdk1 (MW:15.61 kDa) and WcNdk2 (MW: 17.82 kDa) were analyzed using antibodies specifically raised against each protein. No WcNdk1 or WcNdk2 protein could be detected by Western blot of the *W. chondrophila*-infected cells at 3 and 8 hpi (Fig. 3B and C). Both proteins became detectable by 24 hpi and reached their highest signal intensity at this time point after normalization to bacterial number, followed by a drop to roughly 30% of that level by 48 hpi. To further assess protein expression at early stages, we performed immunofluorescence microscopy. In contrast to Western blot results, WcNdk1 and WcNdk2 signals were already detectable from 0 to 16 hpi, colocalizing with *W. chondrophila* (Supplementary Fig. S1, antibody specificity was validated as shown in Supplementary Fig. S5). Together, these results suggest that both WcNdk1 and WcNdk2 are expressed throughout the developmental cycle, with an early transcriptional peak.

### 3.4 Subcellular localization of WcNdk1 and WcNdk2 upon *C. trachomatis* heterologous expression

**Fig. 4:**
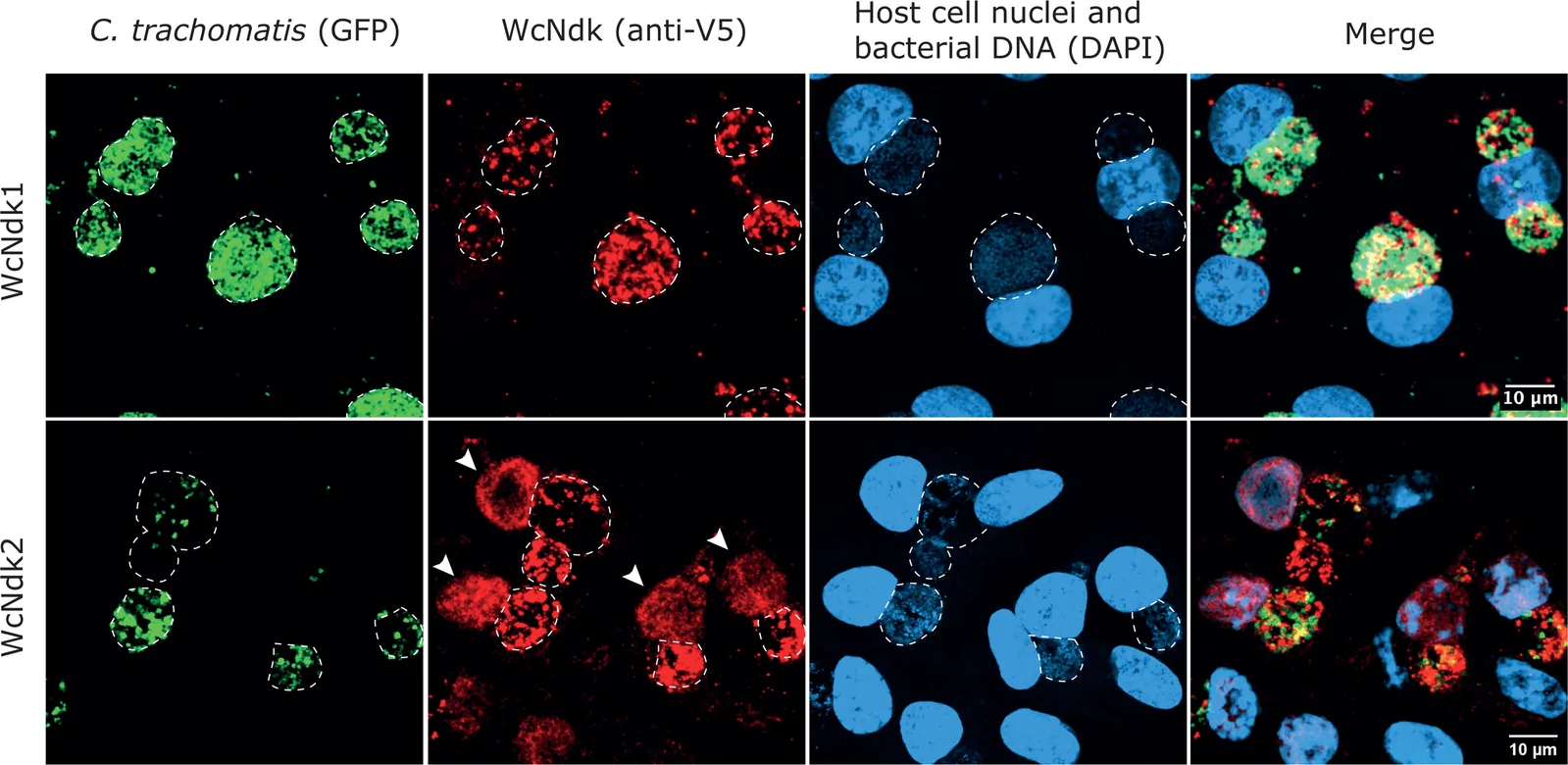
Subcellular localization of WcNdk1 and WcNdk2 overexpressed in C. trachomatis. C. trachomatis was transformed with plasmids expressing either WcNdk1 or WcNdk2 tagged with a V5 epitope. Infected McCoy cells were fixed at 24 hpi. GFP (green) indicates transformed C. trachomatis, anti-V5 (red) detects WcNdk proteins, and DAPI (blue) stains host cell nuclei and bacterial DNA. Dashed white outlines indicate chlamydial inclusions. White arrows point to V5 signal within host nuclei.

While signal peptides are typically associated with protein secretion, previous studies have demonstrated that Ndk can be secreted despite lacking a canonical signal sequence (Kamath et al., 2000; Neeld et al., 2014; Atanasova et al., 2016; Bunce and Khanim, 2018). Moreover, because both WcNdk1 and WcNdk2 are experimentally demonstrated to be secreted into the host cytoplasm (Kebbi-Beghdadi et al., 2019; 2020), the WcNdk2 signal peptide may not strictly dictate initial bacterial secretion. Instead, the presence of this signal peptide suggests that WcNdk2 is subsequently translocated to a specific host subcellular compartment, thereby suggesting a distinct biological role compared to WcNdk1. To assess this possibility and in absence of a genetic system to modify *W. chondrophila,* we used *C. trachomatis* as a heterologous system to overexpress V5-tagged versions of WcNdk1 and WcNdk2, from the inducible shuttle vector pGL2, and studied their subcellular localization by immunofluorescence and confocal microscopy (Fig. 4). In this heterologous expression assay, WcNdk1 was exclusively detected inside the bacteria-containing vacuole, further called the inclusion, closely associated with *C. trachomatis*, and no signal was observed in the host cell cytoplasm. Although this does not rule out secretion of WcNdk1, it suggests limited accumulation or detection outside the inclusion. In contrast, WcNdk2 was detected both in chlamydial inclusions and host cell nucleus, implying that it may be transported into the nucleus, despite the absence of a predicted nuclear localization signal (prediction by DeepLoc-2.1 and SPSignal). Nuclear fractionation analysis further supported nuclear association of WcNdk2 in this system (Supplementary Fig. S3). Furthermore, deletion of the predicted signal peptide restricted WcNdk2 to the inclusion (Supplementary Fig. S6A), suggesting that the N-terminal signal peptide, or at least part of it, was still present on the mature secreted protein and contributed to WcNdk2 trafficking toward host cell nucleus. To further investigate the nuclear localization of WcNdk2, we cloned the gene into the pBOMBL vector (Ouellette et al., 2021) and overexpressed the protein in *C. trachomatis*. Upon infection of McCoy cells with the transformed bacteria, inclusions appeared smaller compared to controls. Due to these growth defects, reliable localization of WcNdk2 could not be realized in this system. This vector-associated toxicity likely reflects bacterial stress caused by excessive or mis-regulated expression of WcNdk2, similar to the overexpression-associated toxicity reported for type III effectors in *Pseudomonas aeruginosa* (Yahr & Wolfgang, 2006). Consistently, WcNdk2 expression was also not detected in the *Yersinia enterocolitica* type III secretion assay, suggesting poor tolerance or instability of the protein in this system.

### 3.5 Subcellular localization of WcNdk1 and WcNdk2 in transfected cells

**Fig. 5:**
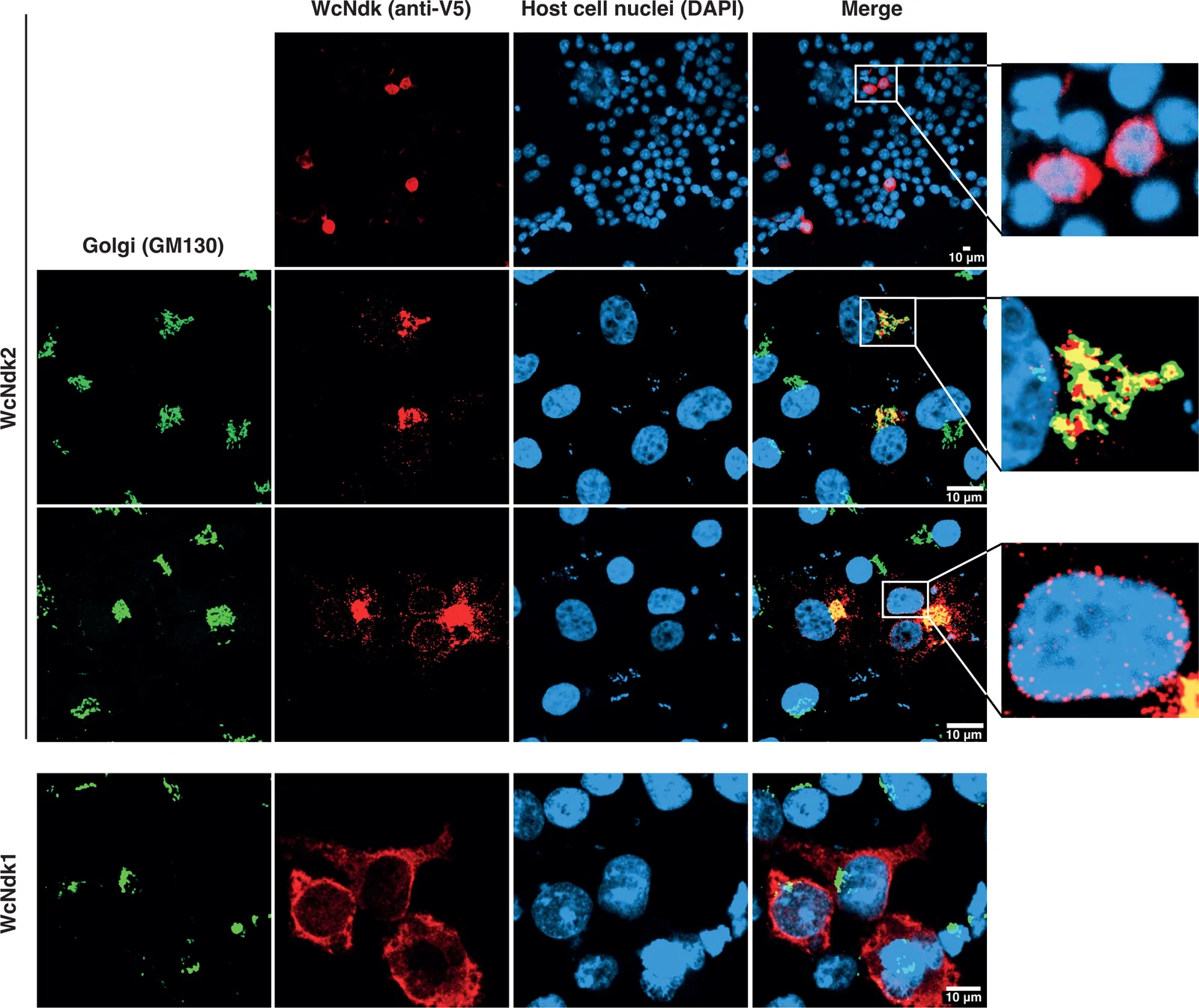
Subcellular distribution of WcNdk1 and WcNdk2 in HEK293T. HEK293T cells were transiently transfected with C-terminal V5-tagged WcNdk1 and WcNdk2 and stained with the Golgi marker GM130 (green), anti-V5 antibody (red), and DNA (DAPI, blue). Nuclear and prenuclear-Golgi localizations of WcNdk2 are mutually exclusive and were not observed simultaneously within the same cell. High-magnification insets highlight the co-localization of WcNdk2 (red) with the host nucleus (blue), Golgi (green) or the perinuclear region.

To further confirm the observed subcellular localization of WcNdk1 and WcNdk2 in another heterologous system, we transiently expressed the V5-tagged WcNdk1 and WcNdk2 in HEK293T cells and analyzed their distribution by immunofluorescence microscopy. WcNdk1 was found exclusively in the cytoplasm and was absent from cell compartments such as nucleus or Golgi (Fig. 5). WcNdk2 showed two mutually exclusive distribution patterns. In some cells the V5 signal was found throughout the nucleus, while in others, it was localized to perinuclear-Golgi regions, with no nuclear signal (Fig. 5). A similar nuclear localization of WcNdk2 was also observed in transfected HeLa cells (Supplementary Fig. S2), indicating that this targeting is not cell-type specific. Additionally, a truncated version of WcNdk2 lacking the signal peptide was not directed to the nucleus, suggesting that this sequence, or at least part of it, functions as a nuclear targeting signal (Supplementary Fig. S6B). Moreover, fusing the WcNdk2 signal peptide to mCherry redirected the fluorescent reporter to the Golgi and perinuclear regions, contrasting with the diffuse intracellular signal observed with mCherry alone (Supplementary Fig. S6C).

### 3.6 The Ndk inhibitor azidothymidine inhibits the growth of *W. chondrophila*

**Fig. 6:**
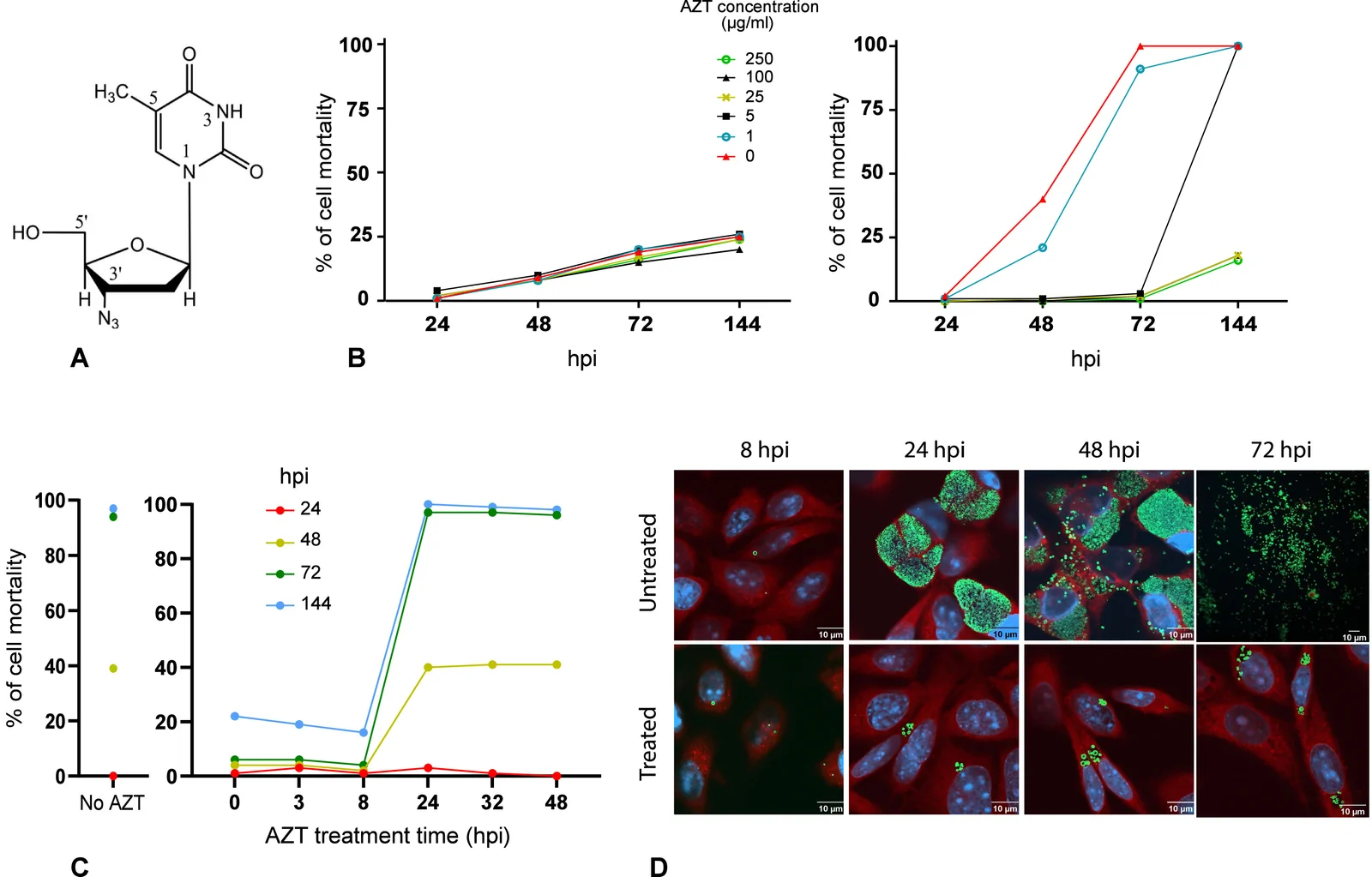
AZT inhibits W. chondrophila growth in infected host cells. (A) Chemical structure of azidothymidine (AZT). (B) AZT treatment of uninfected host cells, serving as a negative control (left panel) and treatment of infected host cells at concentrations ≥25 μg/mL (right panel). (C) Effect of AZT addition on W. chondrophila-infected cells, showing reduced cell mortality when AZT was added 0 to 8 hpi. (D) Confocal microscopy of W. chondrophila-infected cells treated or not treated with AZT. In untreated conditions, W. chondrophila formed typical intracellular inclusions, whereas AZT treatment resulted in enlarged, aberrant structures. Bacteria are labeled using anti-W. chondrophila antibody (green); host cell nuclei are stained with DAPI (blue); and the host structures are marked with Concanavalin A (red). Data shown are from a representative experiment; independent experiments yielded comparable results.

Since *W. chondrophila* is currently genetically intractable, we employed chemicals to inhibit Ndk function in this pathogen. Azidothymidine (AZT) is a known Ndk inhibitor (Gallois-Montbrun et al., 2002; Schaertl et al., 1998; Valenti et al., 1999; Wambaugh et al., 2017; Wang et al., 2019; Xu et al., 1997) structurally similar to thymidine, but where the 3’-hydroxyl group on the deoxyribose sugar is replaced by an azido group (N_3_) (Fig. 6A). In *Aspergillus flavus* AZT inhibits Ndk enzymatic activity by forming a strong hydrogen bond with key active site residues (Arg-104, His-117 and Asp-120) (Wang et al., 2019). Since these three residues are highly conserved in WcNdk1 and WcNdk2, we hypothesized that AZT could also serve as an effective inhibitor of Ndks in *W. chondrophila* (Fig. 2A).

To test this hypothesis, we treated both uninfected and *W. chondrophila*-infected McCoy cells with increasing concentrations of AZT following infection and measured cell death resulting from bacterial proliferation using propidium iodide (PI) staining. In the absence of AZT or at low concentrations (1 µg/ml), *W. chondrophila*-infected cultures exhibited a sharp increase in cell mortality over time, reaching maximal levels by 144 hpi. Treatment with AZT at concentrations above 25 µg/ml significantly suppressed infection-induced cell death. A delay in cell mortality was observed with the intermediate concentration of 5 µg/ml (Fig. 6B, right panel). In contrast, in uninfected cells, cell lysis remained comparable across all AZT concentrations, showing no significant difference from the no-AZT control. Therefore, the slight increase in cell death observed over time in untreated cultures is due to normal cell aging and turnover, and not to AZT treatment (Fig. 6B, left panel). These results indicate that AZT protected against *W. chondrophila*-induced cell death in a dose-dependent manner.

### 3.7 Only early AZT treatment prevents *W. chondrophila*–mediated host cell death

To define the timing of AZT inhibitory effect, *W. chondrophila*-infected McCoy cells were treated with 25 µg/ml AZT at 0, 3, 8, 24, 32, or 48 hpi and the PI uptake was monitored at 24, 48, 72, and 144 hpi (Fig. 6C). When AZT was added at 0, 3, or 8 hpi, cell mortality remained minimal (approximately 25% at 144 hpi), comparable to uninfected controls, indicating complete protection against bacterial‐induced cell death. In contrast, administering AZT at 24 hpi or later failed to prevent cell death. This result indicates that once RB replication and inclusion expansion are established, AZT cannot reverse the course of infection.

### 3.8 AZT induces the production of ABs in *W. chondrophila*

We performed confocal microscopy on infected McCoy cells treated with 25 µg/ml AZT at 0 hpi, to assess the morphology of *W. chondrophila* in presence of AZT. In untreated cultures, *W. chondrophila* underwent normal intracellular development ending with host cell lysis. At 8 hpi, EBs were observed attached to the host cell surface and internalized in both AZT-treated and untreated cultures indicating that AZT does not interfere with bacterial attachment or entry (Fig. 6D). By 24 hpi, inclusions had formed in both conditions. However, in AZT-treated cells, these inclusions failed to expand, and only a limited number of bacteria were detected within them. This suggests that although EB-to-RB differentiation and initial rounds of replication may occur, bacterial proliferation is subsequently arrested. In addition, RBs appeared slightly enlarged in AZT-treated cultures, consistent with the formation of ABs. These abnormal conditions persisted until 72 hpi, indicating a disruption of the developmental cycle likely resulting in bacterial persistence.

### 3.9 Effect of AZT on *W. chondrophila* growth is probably correlated with WcNdk2 activity

**Fig. 7:**
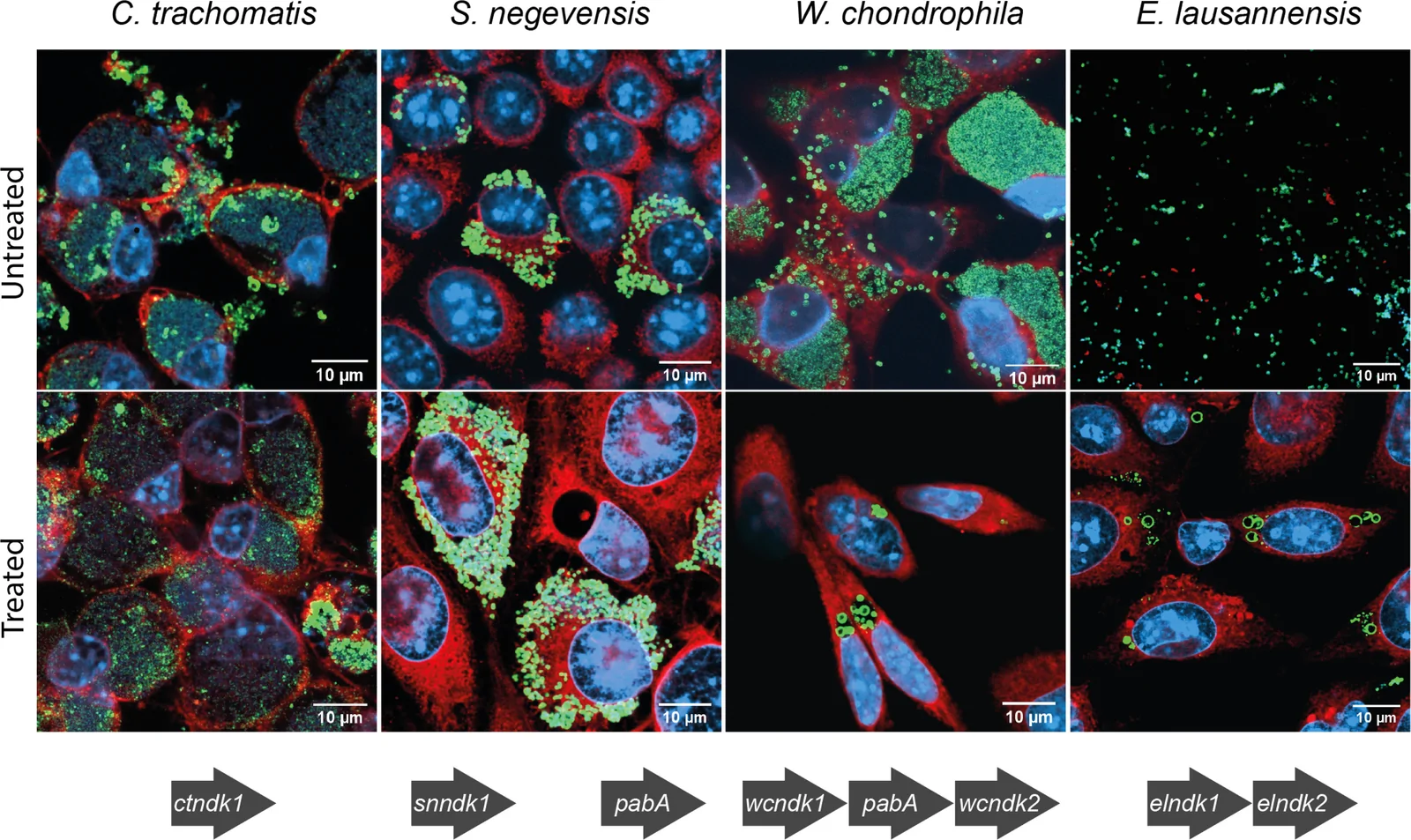
AZT sensitivity is restricted to Chlamydiota species encoding ndk2. Representative confocal micrographs of McCoy cells infected with C. trachomatis, S. negevensis, W. chondrophila or E. lausannensis, treated or not treated with 25 μg/ml AZT. The infected cells were fixed and stained 48 hpi. The lower panel shows the ndk operon arrangement: C. trachomatis and S. negevensis, each carry a single ndk gene. S. negevensis retains pabA elsewhere in the genome. W. chondrophila carries the two wcndk genes with a pabA in the middle. E. lausannensis encodes a two-gene elndk1–elndk2 operon but lacks pabA. Bacteria are stained with species-specific antibodies. Host cytoplasm is labeled with Concanavalin A (red), and nuclei are stained with DAPI (blue). ctndk: C. trachomatis ndk1, snndk1: S. negevensis ndk1, elndk1: E. lausannensis ndk1, elndk2: E. lausannensis ndk2.

*W. chondrophila* encodes two *ndk* paralogs, probably organized in a single operon, with *pabA* positioned between them. To investigate which *ndk* paralog mediates susceptibility to AZT, and whether this susceptibility is linked to the presence of the *pabA* gene, we selected representative *Chlamydiota* species with different *ndk* gene cluster configurations and compared their response to AZT treatment. The selected species include *C. trachomatis*, which carries a single *ctndk* gene and lacks *pabA; Simkania negevensis,* which also has a single *snndk* gene but retains *pabA* elsewhere in the genome; *Estrella lausannensis*, which harbors a two-genes cluster *elndk1*–*elndk2* without *pabA*; and *W. chondrophila*, which contains the full *wcndk1*–*pabA*–*wcndk2* regulon (Fig. 7, bottom panel). To evaluate the effect of AZT on these species, infected McCoy cells were treated with 25 µg/ml at 0 hpi and fixed at 48 hpi before immunostaining and observation under confocal microscopy. As shown in Fig. 7, *C. trachomatis* and *S. negevensis* exhibited normal intracellular development in presence of AZT, with no significant growth inhibition. In contrast, *E. lausannensis*, like *W. chondrophila*, displayed enlarged intracellular structures resembling ABs. This suggests either that AZT inhibitory effect depends on the presence of *ndk2*, regardless of the presence of *ndk1* and *pabA* or that susceptibility is due to the presence of the 2 paralogs.

## 4 Discussion

*Waddlia chondrophila* encodes two *ndk* paralogs that were shown to be preferentially expressed in RBs over EBs (Ardissone et al., 2020). Although Ndks are highly conserved in prokaryotes, their function in members of the phylum *Chlamydiota* is not well characterized and we hypothesized that they could play an important role during chlamydial development. Here, we show that both *wcndk1* and *w*c*ndk2* are transcribed early during infection, leading to protein accumulation at mid-cycle. Heterologous expressions of WcNdk1 and WcNdk2 revealed differential localization, suggesting potentially divergent roles. Noteworthy, the Ndk inhibitor AZT restricted growth only in *Chlamydiota* members harboring both *ndk* paralogs, including *W. chondrophila* and *E. lausannensis*.

The diverse *ndk* paralogs number within members of the *Chlamydiota* reveals lineage-specific adaptations with possible metabolic and pathogenic consequences. While most *Chlamydiota* species possess a single *ndk* gene, a subset, including members of the *Parachlamydiaceae, Waddliaceae*, and *Criblamydiaceae*, harbor a second paralog (*ndk2*), likely arising from a gene duplication event. This duplication may have provided a selective advantage by increasing metabolism flexibility, which may account for the faster growth of *W. chondrophila* and *E. lausannensis* in mammalian cells as compared to other members of the phylum such as *C. trachomatis* or *S. negevensis* (Kebbi-Beghdadi et al., 2011). On the other hand, the absence of the second paralog in *Rhabdochlamydiaceae, Simkaniaceae* and *Chlamydiaceae* coincides with their adaptations to more specific host niches and reduced metabolic pathways. The *pabA* gene is also absent in all members of the *Chlamydiaceae* family. In this family, enzymes from other biosynthetic pathways meet folate requirements (Adams et al., 2014). The mechanisms driving the evolution of this gene cluster in chlamydial families remain unknown.

The temporal expression patterns of *wcndk1* and *wcndk2* provide insights into their potential biological roles and regulation during the *W. chondrophila* developmental cycle. Their synchronized expression profiles are in line with their potential organization within a single operon. The apparent mid-cycle increase in Ndk protein at 24 hpi coincides with maximal bacterial replication, elevated demand for NTPs, and enhanced trafficking of host-derived metabolites to the inclusion during intracellular growth.

Both *W. chondrophila ndk* paralogs retain the universally conserved HGSD active‐site motif and surrounding hydrophobic residues essential for autophosphorylation and phosphotransferase activities. This structural conservation points to the possibility that WcNdk1 and WcNdk2 could potentially involve phosphorylation mechanisms in their respective roles. Despite sharing conserved kinase domains, WcNdk1 and WcNdk2 showed significant differences in their subcellular localization. This divergence suggests functional specializations and may indicate that the two proteins utilize divergent cellular trafficking mechanisms during infection.

In the *C. trachomatis* expression system, WcNdk1 was confined to the bacteria-containing vacuole. This restricted distribution suggests that WcNdk1 may primarily serve bacterial intracellular functions. However, a previous study provided evidence for the secretion of WcNdk1 into the host cell cytosol (Kebbi-Beghdadi et al., 2020; Kebbi-Beghdadi et al., 2019). It is therefore possible that WcNdk1 is secreted transiently or at levels insufficient to be detected via immunofluorescence. In contrast, WcNdk2 exhibited nuclear localization, which was also observed upon transfection in HEK293T or HeLa cells and was dependent on the presence of the signal peptide. In addition, the markedly lower number of cells expressing WcNdk2 compared with those expressing WcNdk1 or the signal peptide-deleted WcNdk2 variant suggests that WcNdk2 may exert a toxic effect on host cells when targeted to the nucleus.

WcNdk2 nuclear localization is consistent with previous studies on other pathogens, where Ndk was shown to bind host DNA and regulate gene expression through DNA cleavage (Kumar et al., 2005; Levit et al., 2002). Such putative nuclear activity suggests a potential role for WcNdk2 in reprogramming host transcriptional responses to favor bacterial survival or pathogenicity. Identifying the specific host genes targeted by WcNdk2 represents a critical next step that could uncover novel mechanisms of host manipulation by *W. chondrophila* and provide broader insights into its pathogenesis.

When expressed in HEK293T cells, WcNdk2 localized not only to the nucleus but also to the perinuclear-Golgi region. Bacterial Ndks, such as *Porphyromonas gingivalis* Ndk, are found in the perinuclear area of the host cells. This localization has been linked to the modulation of host purinergic signaling via P2X₇ receptors (Atanasova et al., 2016), implying that the perinuclear localization of WcNdk2 could be a biologically relevant phenomenon rather than nonspecific aggregation. Golgi localization represents a novel observation not previously reported for any bacterial species. One plausible explanation for an association of WcNdk2 with the Golgi is that, after translocation into the host cytosol, WcNdk2 enters the classical ER–Golgi secretory pathway and is packaged into Golgi‐derived vesicles to hydrolyze host extracellular ATP and subvert purinergic signaling. This hypothesis is in line with the prediction that WcNdk2 signal peptide will target the protein to the ER/Golgi/secretory pathway in eukaryotic cells. Another possible explanation is that Golgi recruitment of WcNdk2 might play a role in modulating vesicle trafficking between Golgi and the bacterial inclusion. Although *W. chondrophila* does not redirect sphingomyelin transport from the Golgi to its inclusion (Dille et al., 2015), it may still influence other aspects of vesicular trafficking, altering vesicle formation or fusion processes, to benefit bacterial survival or replication..

AZT is a well-established inhibitor of Ndk (Gallois-Montbrun et al., 2002; Schaertl et al., 1998; Valenti et al., 1999; Wambaugh et al., 2017; Wang et al., 2019; Xu et al., 1997). In our study, AZT treatment of *W. chondrophila*-infected cells protected host cells from bacteria-induced cell death and arrested bacterial growth during the replication phase. Given the conserved nature of Ndk enzymes, both WcNdk1 and WcNdk2 may be biochemically susceptible to AZT. However, our localization data showing that WcNdk2 is detected in host cell compartments when expressed in heterologous systems suggest that this paralog may be more easily exposed to AZT and thus may represent the more biologically-relevant target, thereby explaining why AZT treatment preferentially affects *Chlamydiota* species encoding an Ndk2 paralog. Nevertheless, direct biochemical inhibition of WcNdk2 by AZT remains to be experimentally demonstrated. Our results further showed that AZT must be applied before 24 hpi, to fully prevent *W. chondrophila*–induced cytotoxicity. This early phase of infection coincides with maximum RB replication, inclusion expansion, and active vesicular nutrient trafficking from the Golgi and ER (Croxatto & Greub, 2010). In contrast, WcNdk1, which predominantly localizes within the bacteria, and is likely involved in maintaining bacterial nucleotide pools, appears less susceptible to AZT, possibly due to limited drug access to the bacteria-containing compartment. These findings collectively support a model in which the inhibition of host-targeted WcNdk2 plays the key role in AZT antimicrobial activity against *W. chondrophila*.

Although localization studies using heterologous expression systems suggest that WcNdk2 may interact with host cells, the possibility of overexpression artifacts or mis-localization cannot be entirely ruled out. Furthermore, these specific localizations could not be directly observed during *W. chondrophila* infection, likely due to transient translocation or low protein abundance, a technical challenge frequently encountered with secreted bacterial effectors. Nevertheless, we previously bypassed these imaging limitations and successfully demonstrated the secretion of WcNdk2 via mass spectrometry (Kebbi-Beghdadi et al., 2019).

An additional limitation of our study is that AZT, despite being widely used as an Ndk inhibitor, may exert pleiotropic effects in bacteria, and we currently lack direct *in vitro* ATPase assays confirming WcNdk2 as the molecular target of AZT.

**Fig. 8:**
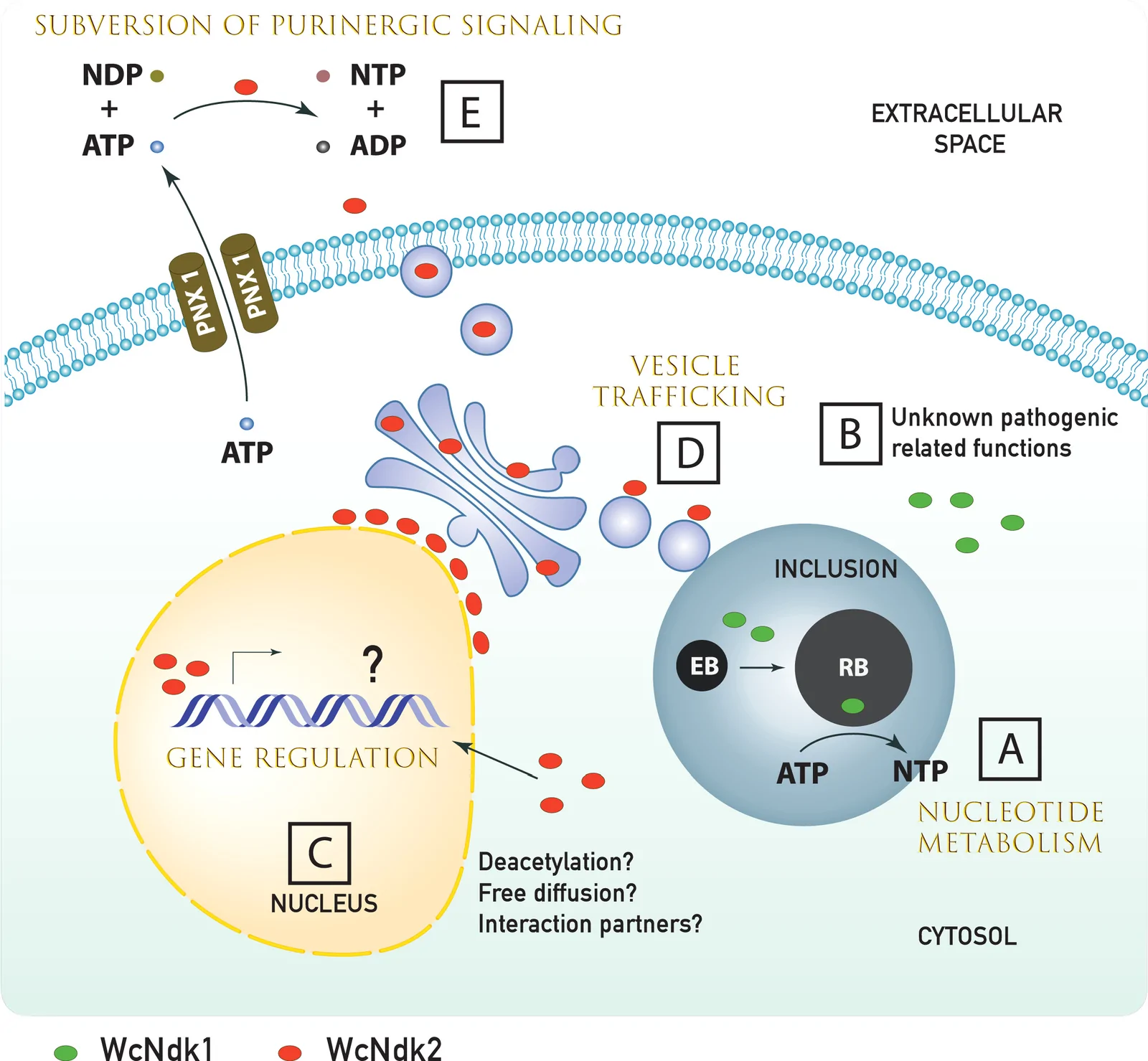
Hypothetical model summarizing potential roles of WcNdk1 and WcNdk2 of W. chondrophila. This schematic illustrates a conceptual model that integrates the localization patterns observed in this study with functions previously described for Ndk in other bacteria. The proposed roles are speculative and are not experimentally demonstrated in W. chondrophila. A: WcNdk1 is predominantly retained within the bacterial inclusion and bacteria, where it may support nucleotide metabolism and housekeeping functions. B: A fraction of WcNdk1 may reach the host cytosol, however, the specific functions of cytosolic WcNdk1 remain undefined. C: WcNdk2 shows nuclear-associated localization in heterologous systems suggestingpossible effect on host gene regulation, althoughspecific targets remain unknown. D: Golgi/perinuclear localization of WcNdk2 may reflect vesicle trafficking and possible acquisition of host-derived metabolites required for inclusion expansion and bacterial survival. E: Extracellular WcNdk2 may modulate purinergic signaling by reducing extracellular ATP availability through Ndk-mediated phosphotransfer reactions.Illustration generated using Adobe Illustrator 2024 (Adobe Inc., USA).

Based on our findings in the present study and on functions previously described for Ndks in other bacteria, we propose a hypothetical working model summarizing potential roles of the two W. chondrophila Ndks (Fig. 8). However, the lack of genetic manipulation tools in *W. chondrophila* currently impedes direct investigation of Ndk function via gene deletion or mutagenesis. In absence of such studies, the proposed roles remain speculative and should be regarded as a basis for future studies rather than a definitive functional characterization.

## 5 Conclusions

Given its diverse activities, Ndk has attracted interest across multiple disciplines, including microbiology, cell biology, and drug development. In this study, we propose chlamydial Ndk as a multifunctional protein with roles extending beyond nucleotide metabolism, highlighting its potential involvement in host–pathogen interactions. Future studies will aim at identifying the eukaryotic partner(s) of WcNdk2 as well as developing genetic tools to directly investigate WcNdks function. The present findings not only enhance our understanding of Ndk biology in representatives of the *Chlamydiota* phylum but also point to Ndk2 as a potential therapeutic target, opening new avenues for dissecting host manipulation strategies in obligate intracellular bacteria.

## Funding

This work was supported by the Swiss National Science Foundation (SNSF) [Grant No. 197768].

## CRediT authorship contribution statement

**Giti Ghazi-Soltani:** Conceptualization, Methodology, Investigation, Formal analysis, Visualization, Writing – original draft. **Carole Kebbi-Beghdadi:** Investigation, Validation, Resources, Writing – review & editing. **Simone E. Adams:** Validation, Writing – review & editing. **Gilbert Greub:** Supervision, Conceptualization, Project administration, Funding acquisition, Writing – review & editing.

## Declaration of competing interest

The authors declare that they have no known competing financial interests or personal relationships that could have appeared to influence the work reported in this paper.

## Data Availability

Data supporting the findings of this study are available from the first author (Giti Ghazi-Soltani) upon reasonable request.

## Appendix Supplementary materials

Image, application 1

Image, application 2

Image, application 3

Image, application 4

Image, application 5

Image, application 6

Image, application 7

Image, application 8

Image, application 9
