# Supplementary figures and images for: Functional insights into nucleoside diphosphate kinases encoded by two ndk paralogs in Waddlia chondrophila
Source: Curr Res Microb Sci. 2026 Jun 17;11:100635. doi: 10.1016/j.crmicr.2026.100635 (PMC13318545; doi:10.1016/j.crmicr.2026.100635)

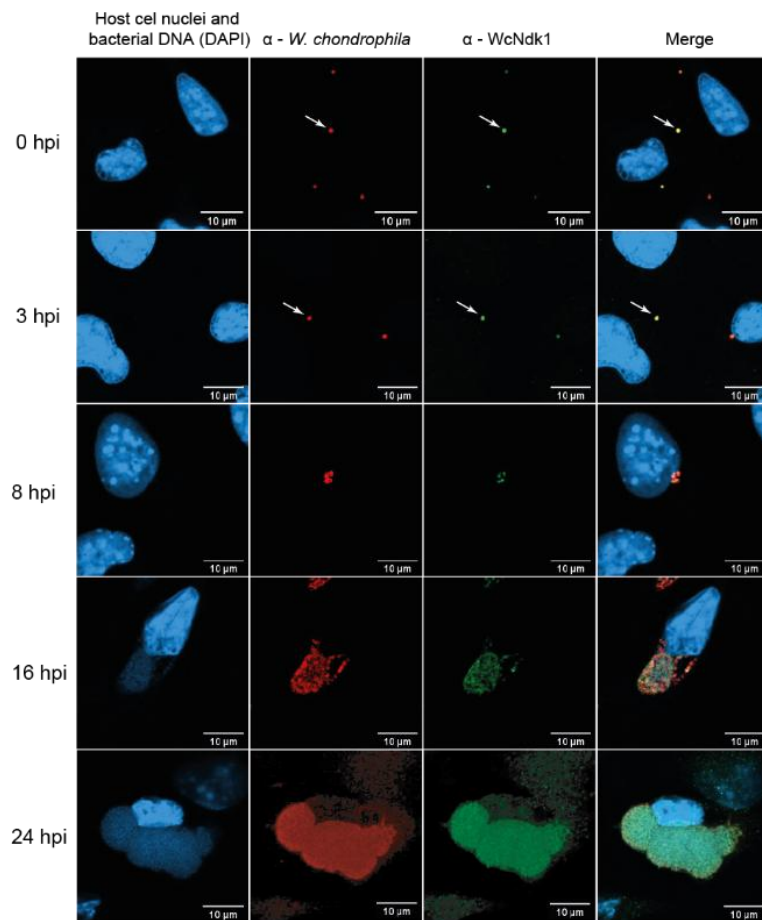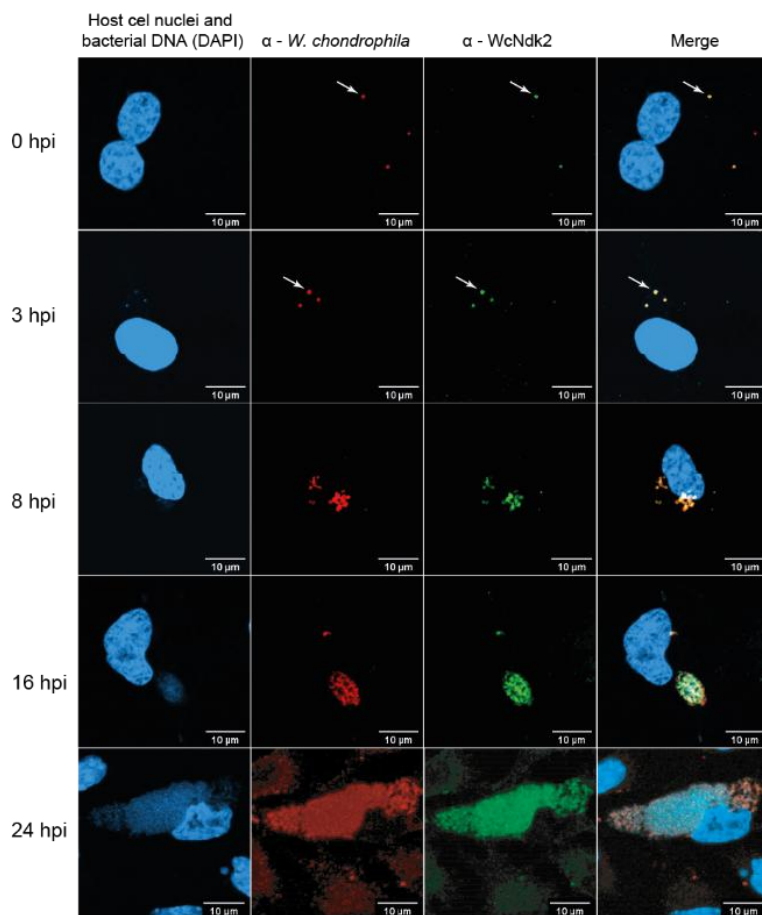

Supplement: Supplementary file 1 [file mmc1.pdf]

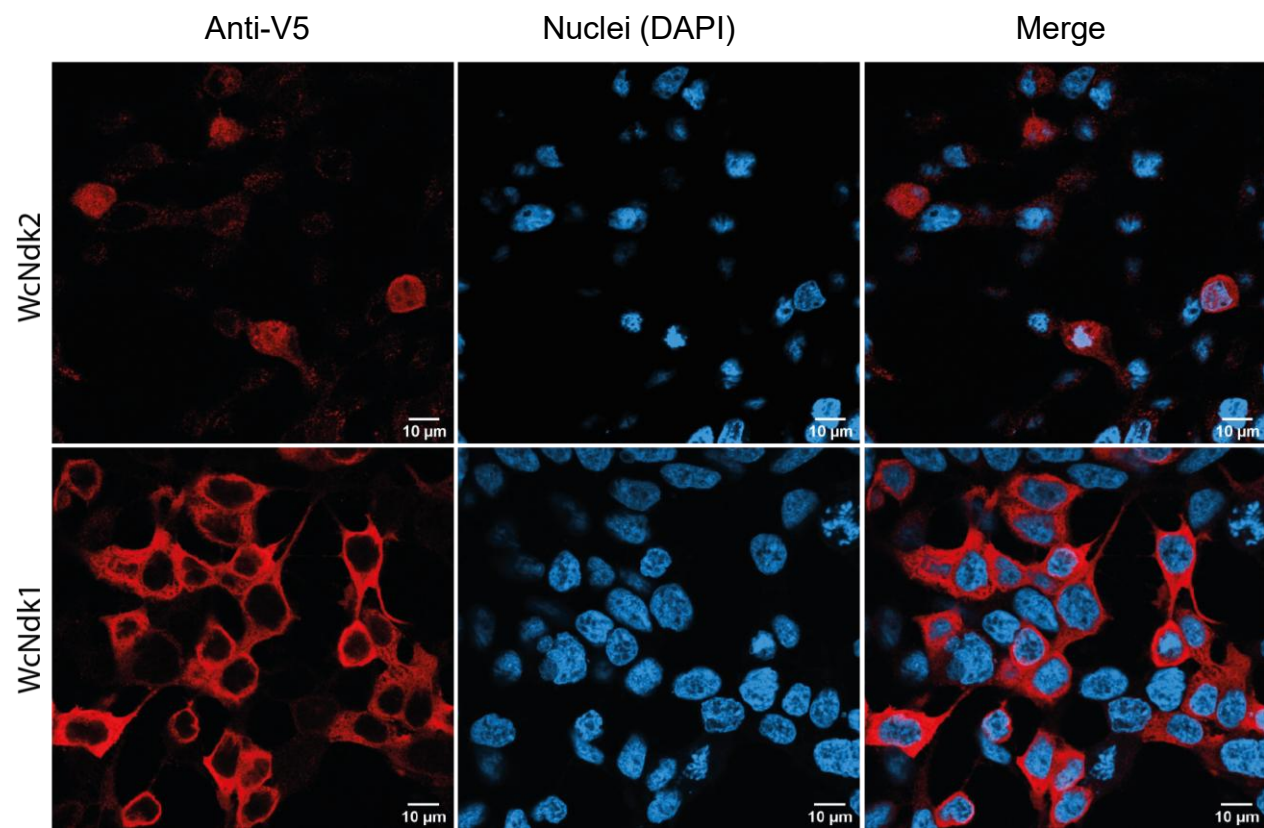

Supplement: Supplementary file 2 [file mmc2.pdf]

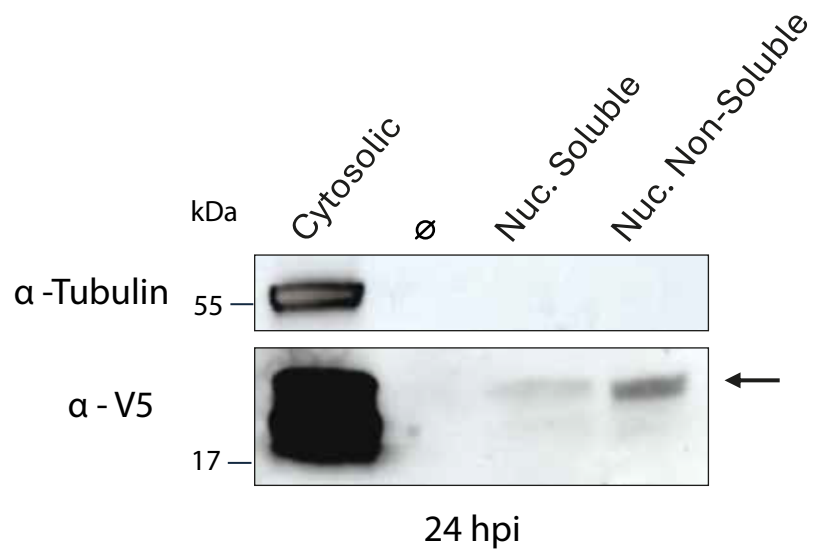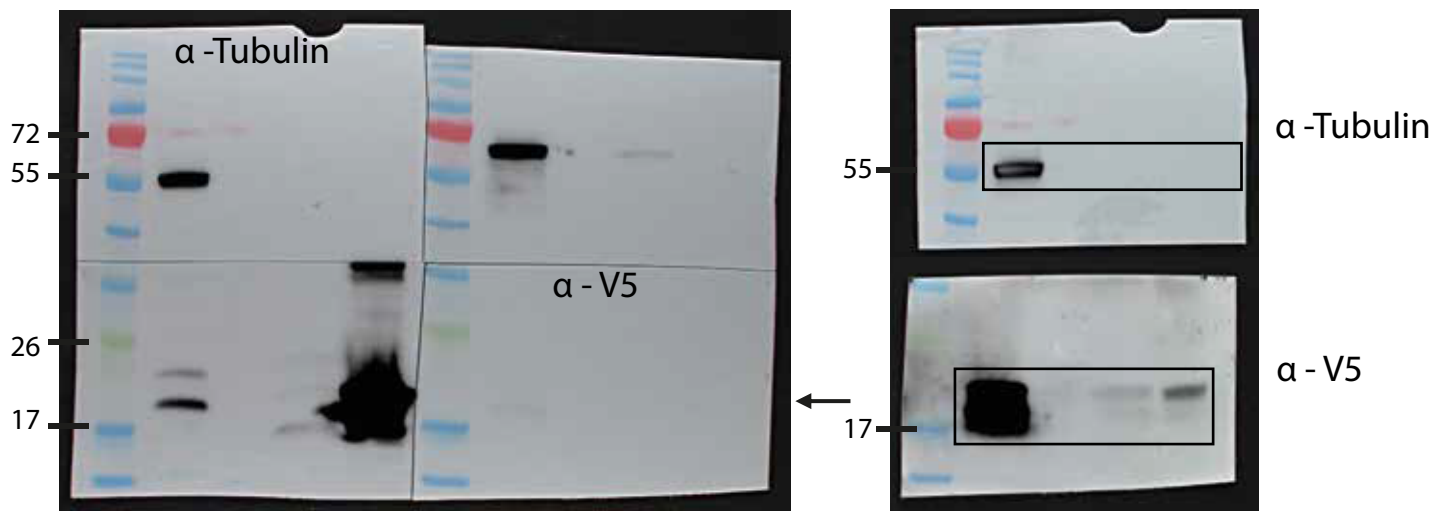

Supplement: Supplementary file 3 [file mmc3.pdf]

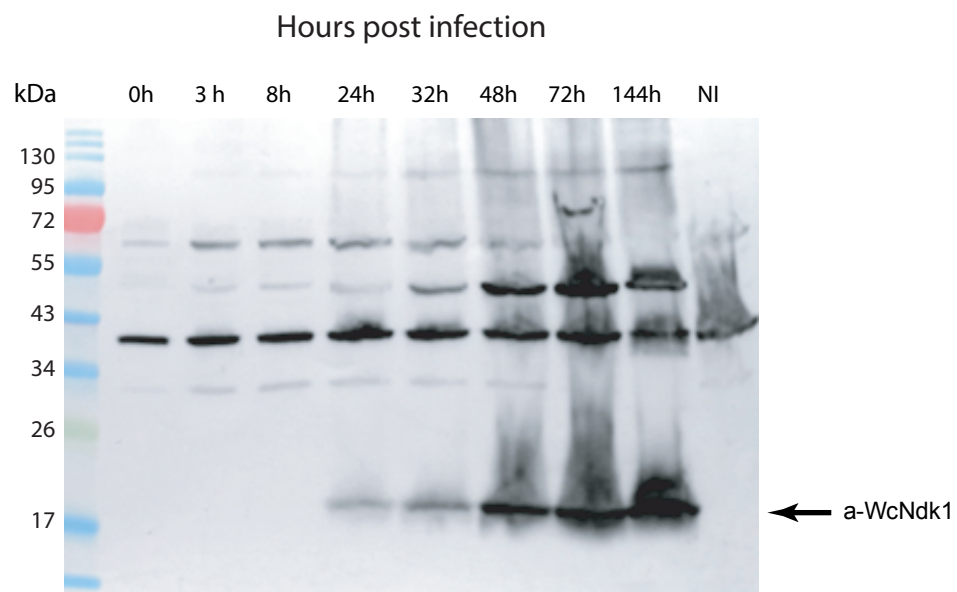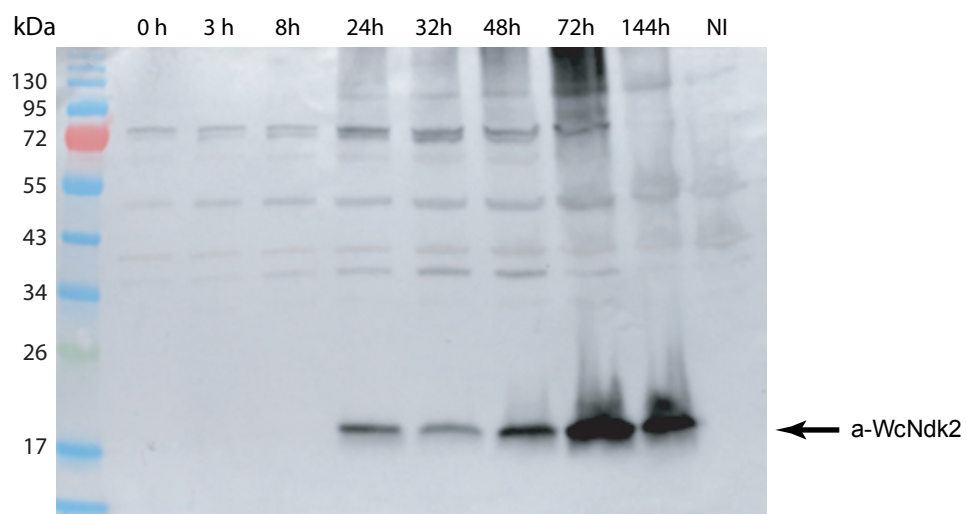

Supplement: Supplementary file 4 [file mmc4.pdf]

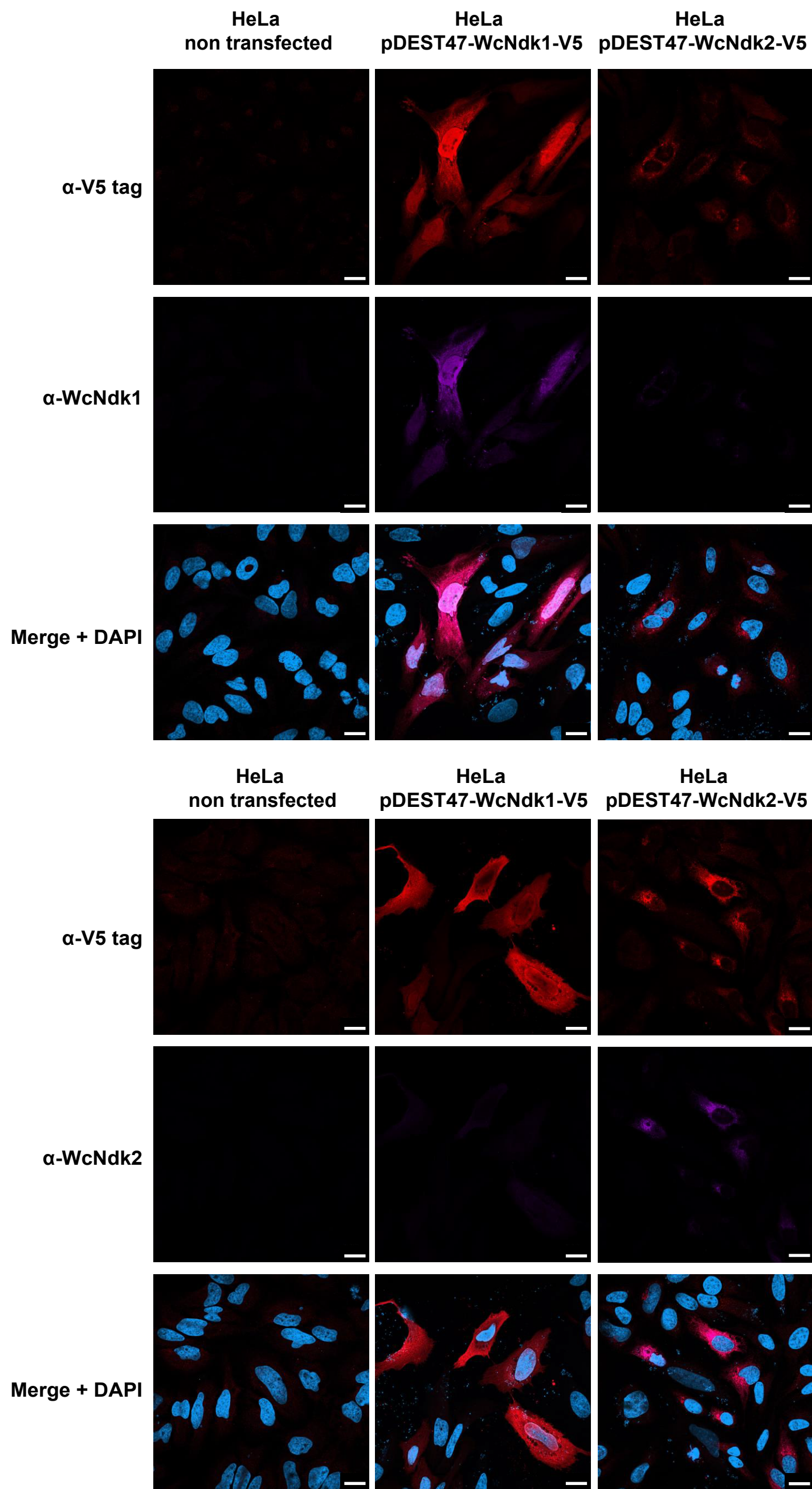

Supplement: Supplementary file 5 [file mmc5.pdf]

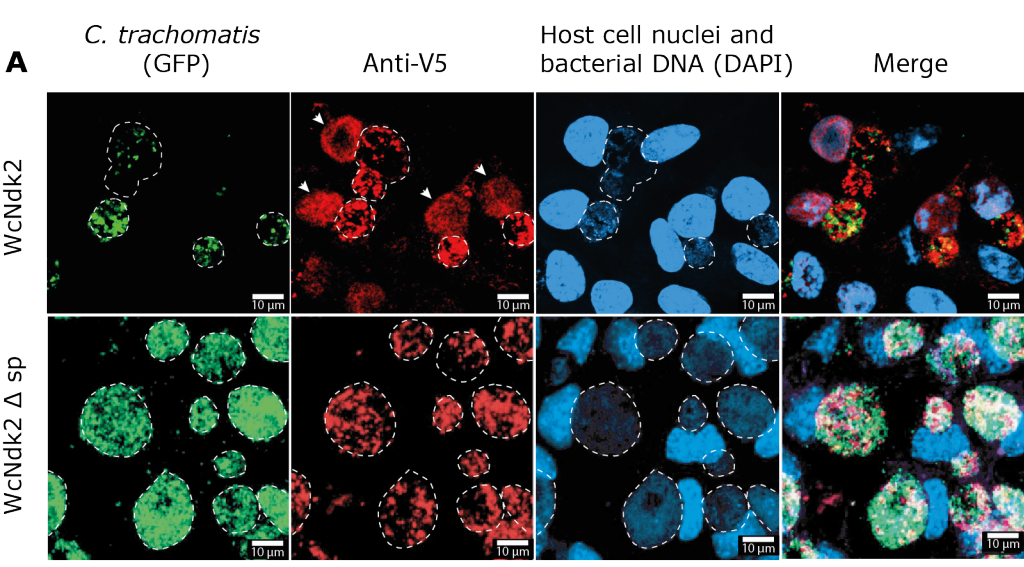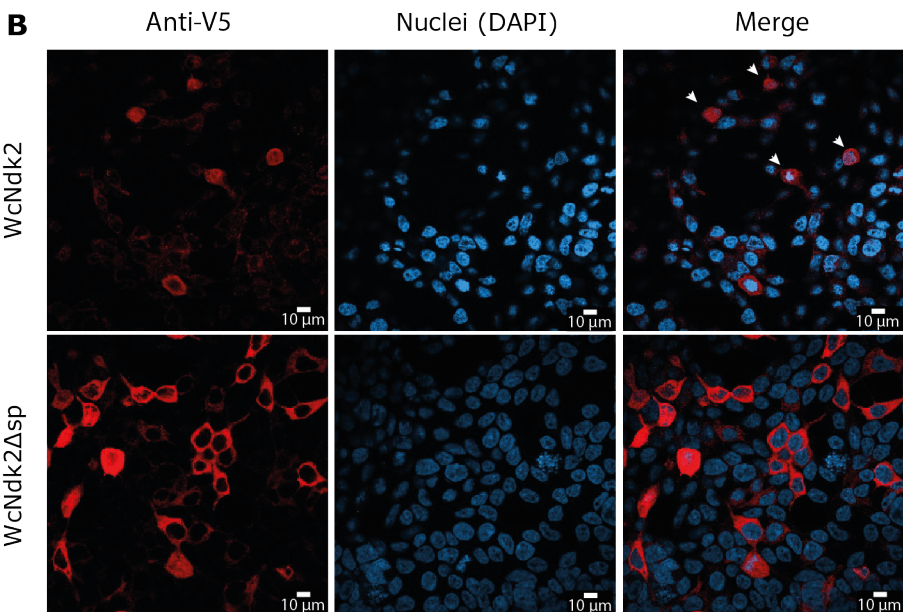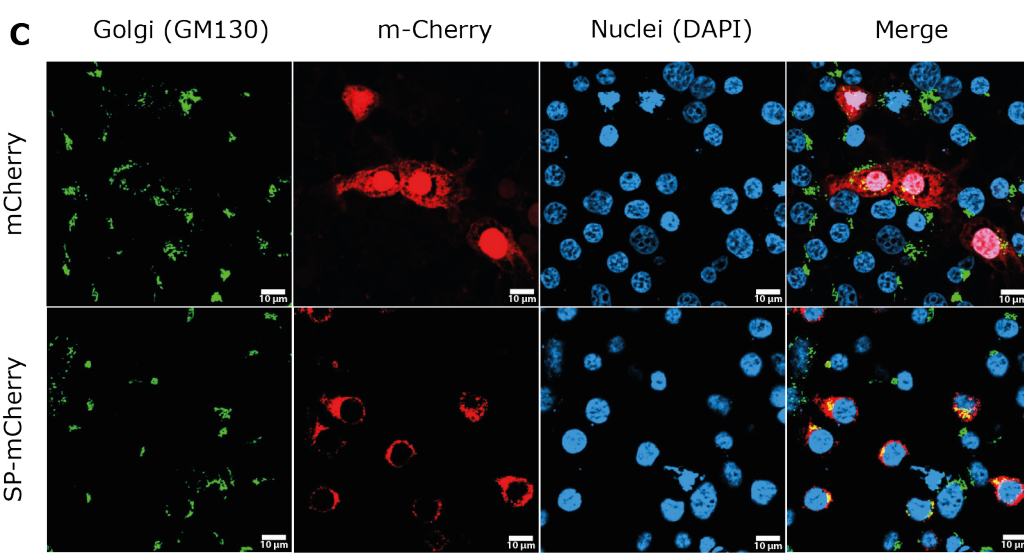

Supplement: Supplementary file 6 [file mmc6.pdf]
