# Supplementary material for: Functional insights into nucleoside diphosphate kinases encoded by two ndk paralogs in Waddlia chondrophila
Source: Curr Res Microb Sci. 2026 Jun 17;11:100635. doi: 10.1016/j.crmicr.2026.100635 (PMC13318545; doi:10.1016/j.crmicr.2026.100635)
Supplement: Supplementary file 7 [file mmc7.pdf]

Target gene	Oligo type	Sequence (5' -> 3')	Label	Amplicon size (bp)
wcw_1543 (*wcndk1*)	Forward primer	tgg-tat-tgg-aag-gag-acg-atg-c	-	160
wcw_1543 (*wcndk1*)	Reverse primer	tcc-gtt-ttt-gcc-gtt-tct-gg	-	160
wcw_1545 (*wcndk2*)	Forward primer	agt-tgt-tgc-gat-ggt-act-gga	-	150
wcw_1545 (*wcndk2*)	Reverse primer	gaa-tct-gat-cca-tgc-acg-gc	-	150
Waddlia 16S rRNA	Forward primer	ggc-cct-tgg-gtc-gta-aag-ttc-t	-	101
Waddlia 16S rRNA	Reverse primer	cgg-agt-tag-ccg-gtg-ctt-ct	-	101
Waddlia 16S rRNA	TaqMan prob	FAM-cAt-ggg-aaC-aag-aga-agG-ATg-BHQ	FAM/BHQ-1	-

Table S1: Primer sequences used for RT-qPCR in this study. Sequence letters in capital are Locked Nucleic acids.
