# Supplementary material for: Functional insights into nucleoside diphosphate kinases encoded by two ndk paralogs in Waddlia chondrophila
Source: Curr Res Microb Sci. 2026 Jun 17;11:100635. doi: 10.1016/j.crmicr.2026.100635 (PMC13318545; doi:10.1016/j.crmicr.2026.100635)
Supplement: Supplementary file 8 [file mmc8.pdf]

	C. sequanensis_Ndk2	E. lausannensis_Ndk2	W. chondrophila_Ndk2	Neochlamydia sp_Ndk2	P. canthamoebae_Ndk2	Neochlamydia sp_Ndk1	C. sequanensis_Ndk1	E. lausannensis_Ndk1	P. acanthamoebae_Ndk1	W. chondrophila_Ndk1	Rabdochlamydia_Ndk	S. negevensis_Ndk	C. trachomatis_Ndk	P.aeruginosa_Ndk	Thioalbus dentrifivans_Ndk	Legionella pneumophila_Ndk	Halofilum ochreceum_Ndk	M. tuberculosis_Ndk	E. coli_Ndk	A.flavu_Ndk	A.niger_Ndk
C. sequanensis_Ndk2	**100%**																				
E. lausannensis_Ndk2	**62%**	**100%**																			
W. chondrophila_Ndk2	**49%**	**56%**	**100%**																		
Neochlamydia sp_Ndk2	**48%**	**52%**	**57%**	**100%**																	
P. canthamoebae_Ndk2	**55%**	**60%**	**62%**	**64%**	**100%**																
Neochlamydia sp_Ndk1	**51%**	**56%**	**55%**	**57%**	**57%**	**100%**															
C. sequanensis_Ndk1	**50%**	**56%**	**62%**	**56%**	**60%**	**77%**	**100%**														
E. lausannensis_Ndk1	**48%**	**57%**	**60%**	**56%**	**57%**	**82%**	**81%**	**100%**													
P. acanthamoebae_Ndk1	**51%**	**57%**	**56%**	**56%**	**56%**	**81%**	**78%**	**83%**	**100%**												
W. chondrophila_Ndk1	**49%**	**52%**	**54%**	**59%**	**53%**	**76%**	**73%**	**79%**	**79%**	**100%**											
Rabdochlamydia_Ndk	**46%**	**50%**	**57%**	**53%**	**52%**	**65%**	**64%**	**66%**	**66%**	**66%**	**100%**										
S. negevensis_Ndk	**51%**	**55%**	**57%**	**54%**	**57%**	**77%**	**73%**	**77%**	**78%**	**76%**	**69%**	**100%**									
C. trachomatis_Ndk	**47%**	**53%**	**59%**	**53%**	**55%**	**71%**	**70%**	**72%**	**70%**	**66%**	**67%**	**69%**	**100%**								
P.aeruginosa_Ndk	**47%**	**52%**	**56%**	**54%**	**54%**	**72%**	**70%**	**78%**	**76%**	**80%**	**68%**	**77%**	**66%**	**100%**							
Thioalbus dentrifivans_Ndk	**48%**	**52%**	**55%**	**56%**	**55%**	**75%**	**73%**	**80%**	**79%**	**82%**	**70%**	**81%**	**68%**	**88%**	**100%**						
Legionella pneumophila_Ndk	**52%**	**54%**	**59%**	**55%**	**58%**	**75%**	**72%**	**76%**	**74%**	**72%**	**69%**	**79%**	**72%**	**77%**	**80%**	**100%**					
Halofilum ochreceum_Ndk	**46%**	**51%**	**55%**	**54%**	**53%**	**71%**	**70%**	**77%**	**76%**	**79%**	**67%**	**77%**	**66%**	**99%**	**88%**	**77%**	**100%**				
M. tuberculosis_Ndk	**38%**	**45%**	**48%**	**45%**	**47%**	**57%**	**55%**	**58%**	**57%**	**57%**	**56%**	**59%**	**54%**	**58%**	**59%**	**58%**	**57%**	**100%**			
E. coli_Ndk	**43%**	**48%**	**49%**	**48%**	**49%**	**66%**	**66%**	**72%**	**68%**	**67%**	**65%**	**68%**	**63%**	**70%**	**71%**	**68%**	**69%**	**56%**	**100%**		
A.flavu_Ndk	**36%**	**37%**	**44%**	**40%**	**41%**	**53%**	**52%**	**54%**	**53%**	**53%**	**51%**	**51%**	**49%**	**49%**	**51%**	**52%**	**49%**	**54%**	**52%**	**100%**	
A.niger_Ndk	**36%**	**37%**	**43%**	**39%**	**41%**	**51%**	**51%**	**52%**	**52%**	**53%**	**49%**	**52%**	**51%**	**49%**	**51%**	**52%**	**49%**	**54%**	**51%**	**89%**	**100%**
Homo sapiens_NME2	**34%**	**36%**	**44%**	**39%**	**39%**	**51%**	**49%**	**55%**	**53%**	**53%**	**50%**	**51%**	**51%**	**51%**	**51%**	**52%**	**51%**	**52%**	**52%**	**73%**	**73%**
