# Supplementary material for: Functional insights into nucleoside diphosphate kinases encoded by two ndk paralogs in Waddlia chondrophila
Source: Curr Res Microb Sci. 2026 Jun 17;11:100635. doi: 10.1016/j.crmicr.2026.100635 (PMC13318545; doi:10.1016/j.crmicr.2026.100635)
Supplement: Supplementary file 9 [file mmc9.docx]

**Figure S1**: Immunofluorescence detection of WcNdk1 and WcNdk2 during *W. chondrophila* infection. DAPI staining (blue) marks host nuclei, anti-*W. chondrophila* (red) labels bacteria, and anti-WcNdk1 or anti-WcNdk2 (green) detect the corresponding proteins. Merged images show colocalization in yellow. Arrows indicate the localization of bacteria, WcNdks, and their colocalization. To control signal specificity, the same experiments were performed in parallel with pre-immune sera and no signal colocalizing with bacteria was detected.

**Figure S2**: HeLa cells expressing WcNdk1-V5 or WcNdk2-V5. Cells were transiently trtransfected withWcNdk1 and WcNdk2 constructs carrying a C-terminal V5 tag.

**Figure S3:** Nuclear fractionation analysis of McCoy cells infected with C. trachomatis expressing WcNdk2-V5. Host cells were subjected to nuclear fractionation at 24 hpi. Cytosolic, nuclear soluble and nuclear non soluble fractions were analyzed by immunoblotting using α-V5 antibodies to detect WcNdk2-V5. α-Tubulin was used as cytosolic marker to assess potential cytosolic contamination of nuclear fractions. The upper panel shows the processed blot used for presentation. WcNdk2-V5 signal (arrow) was detected predominantly in the cytosolic fraction, with additional signal observed in nuclear fractions. The lane labeled “∅” represents an empty lane in which no sample is loaded to avoid potential cross contamination from adjacent wells. The lower panels show the corresponding uncropped blots at standard (left panel) and higher exposure (right panel). The areas inside the box indicate the regions shown in the processed image.

**Figure S4**: Full, uncropped immunoblot corresponding to Figure 3C. Arrows indicate the WcNdk1 and WcNdk2 proteins. Bands inside the black boxes were used for density quantification. Bands with similar molecular weight is not present in non-infected (NI) lysates, consistent with the antibody not detecting the host NDPKs at this molecular weight. Time points 72 hpi and 144 hpi are shown here for completeness but were not included in the main figure and not used for quantification because they fall outside the developmental period analyzed in this study.

**Figure S5**: Validation of anti-WcNdk1 and anti-WcNdk2 antibody specificity. HeLa cells were either non transfected or transfected with pDEST47-WcNdk1-V5 or pDEST47-WcNdk2-V5 constructs. Cells were stained either with anti-V5, anti-WcNdk1 or anti-WcNdk2 antibodies to assess specificity. Anti-WcNdk1 specifically detected WcNdk1-V5 expressing cells and showed no signal in WcNdk2-V5 expressing cells. Conversely, anti- WcNdk2 specifically detected WcNdk2-V5 expressing cells without cross-reactivity in WcNdk1-V5 expressing cells. No signal was observed in non-transfected cells. Nuclei were stained with DAPI. Scale bar: 20μm.

**Figure S6. Contribution of the WcNdk2 signal peptide to intracellular localization in heterologous systems.**

(A) Localization of full-length WcNdk2 and the signal peptide deletion construct (WcNdk2ΔSP) during heterologous expression in *C. trachomatis*. McCoy cells infected with GFP-expressing *C. trachomatis* were fixed and stained using anti-V5 antibodies to detect ectopically expressed proteins (red). Host-cell nuclei and bacterial DNA were stained with DAPI (blue). Dashed white outlines indicate chlamydial inclusions. Full-length WcNdk2 displayed localization within the inclusion together with additional host nuclear signal (arrowheads), whereas WcNdk2ΔSP showed a more restricted inclusion-associated localization pattern and no detectable nuclear-associated signal. Scale bar: 10 µm.

(B) Localization of full-length WcNdk2 and WcNdk2ΔSP following transient expression in HeLa cells. Proteins were detected using anti-V5 antibodies (red) and nuclei were stained with DAPI (blue). Full-length WcNdk2 displayed nuclear and perinuclear localization patterns (arrowheads), whereas deletion of the signal peptide resulted in a predominantly cytoplasmic localization pattern. Scale bar: 10 µm.

(C) Localization of mCherry alone or mCherry fused to the WcNdk2 signal peptide (SP-mCherry) following transient expression in HeLa cells. Cells were stained with the Golgi marker GM130 (green) and nuclei were stained with DAPI (blue). mCherry alone displayed a diffuse intracellular distribution, whereas SP-mCherry showed enrichment in Golgi/perinuclear regions, supporting a role for the WcNdk2 N-terminal signal peptide in intracellular trafficking. Scale bar: 10 µm.

**Table S1**: Primer sequences used for RT-qPCR in this study. Sequence letters in capital are Locked Nucleic acids.

**Table S2**: Sequence identity matrix of Ndk proteins across selected species. Heatmap showing pairwise amino acid identity (%) between Ndk proteins from diverse organisms. *A: Aspergillus, C. trachomatis: Chlamydia trachomatis, C. sequanensis: Criblamydia sequanensis, E. coli: Escherichia coli, E. lausannensis: Estrella lausannensis, M: Mycobacterium, P. acanthamoebae: Parachlamydia acanthamoebae, P. aeruginosa: Pseudomonas aeruginosa, S: Simkania, W: Waddlia*.
